# Supplementary material for: The Effectiveness of Interactive Dashboards to Optimise Antibiotic Prescribing in Primary Care: A Systematic Review
Source: Antibiotics (Basel). 2023 Jan 10;12(1):136. doi: 10.3390/antibiotics12010136 (PMC9854857; doi:10.3390/antibiotics12010136)
Supplement: Supplementary file 1 [file antibiotics-12-00136-s001.zip › Table S1 - List of studies excluded and reasons for their exclusion.pdf]

Table S1. **List of studies excluded and reasons for their exclusion**

n	Authors/year	Title	Reason for exclusion
[1]	Gentry et al., 2019	A 20/20 vision: Successful integration of a prescribing dashboard for outpatient antimicrobial stewardship to target 20% reduction by the year 2020	Conference abstract
[2]	Gonzales et al., 2013	A cluster randomized trial of decision support strategies for reducing antibiotic use in acute bronchitis	Intervention group was a computerized decision support (CDS) strategy integrated into the workflow of an electronic health record (HER). It does not describe a visual analytics tool
[3]	DiBardino et al., 2012	An electronic checklist vs. prompting physicians to decrease empirical antibiotic utilization	Setting: intensive care unit (ICU)<br>Intervention: an EHR-based checklist to a face to-face physician prompter. It does not describe a visual analytics tool
[4]	Rutten et al., 2022	An Electronic Health Record Integrated Decision Tool and Supportive Interventions to Improve Antibiotic Prescribing for Urinary Tract Infections in Nursing Homes: A Cluster Randomized Controlled Trial	Intervention: EHR-integrated decision tool. It does not describe a visual analytics tool
[5]	Gjelstad et al., 2006	Can antibiotic prescriptions in respiratory tract infections be improved? A cluster-randomized educational intervention in general practice - The Prescription Peer Academic Detailing (Rx-PAD) Study NCT00272155	Intervention: The elements of the intervention are discussions within the peer group, collection of individual prescription data, audit based on individual feedback reports, as well as a one-day regional work-shop. It does not describe a visual analytics tool
[6]	Gulliford et al., 2011	Cluster randomized trials utilizing primary care electronic health records: methodological issues in design, conduct, and analysis (eCRT Study).	Intervention is a decision support tool incorporated consultation system, which was activated when the family physician entered a medical code for the

n	Authors/year	Title	Reason for exclusion
			respiratory tract infection. It does not describe a visual analytics tool
[7]	Feldstein et al., 2017	Design and implementation of electronic health record integrated clinical prediction rules (iCPR): a randomized trial in diverse primary care settings	Intervention is a decision support tool incorporated system, the participant then received an alert that, if accepted, would reveal the risk calculator. It does not describe a visual analytics tool.
[8]	Palin et al., 2020	Developing the infrastructure to support the optimisation of antibiotic prescribing using the learning healthcare system to improve healthcare services in the provision of primary care in England.	It is not an experimental study (i.e., No intervention) Mainly data analytics to form dashboards.
[9]	Litvin et al., 2011	Development and implementation of a clinical decision support system to promote judicious use of antibiotics in primary care	Conference abstract
[10]	Linder et al., 2009	Documentation-based clinical decision support to improve antibiotic prescribing for acute respiratory infections in primary care: a cluster randomised controlled trial	Intervention is a decision support tool incorporated the Notes page of the HER. The ARI Smart Form includes 6 components: entry of clinical information; patient data display; diagnosis selection; presentation of treatment options with integrated decision support; printing of patient handouts; and access to supporting medical literature. It does not describe a visual analytics tool
[11]	Gulliford et al., 2019	Electronically delivered interventions to reduce antibiotic prescribing for respiratory infections in primary care: cluster RCT using electronic health records and cohort study	Intervention included a decision support tool were deployed remotely into existing practice software to provide patient information sheets and advice on the positive indications for antibiotic prescription

n	Authors/year	Title	Reason for exclusion
			during consultations for respiratory infection. It does not describe a visual analytics tool
[12]	Blair et al., 2017	Feasibility cluster randomised controlled trial of a within-consultation intervention to reduce antibiotic prescribing for children presenting to primary care with acute respiratory tract infection and cough	Intervention: a within-consultation interactive web-based tool, which also provided a data collection tool. It does not describe a visual analytics tool
[13]	Lesprit & Brun-Buisson, 2008	Hospital antibiotic stewardship	Type of study: narrative review
[14]	Mainous et al., 2013	Impact of a clinical decision support system on antibiotic prescribing for acute respiratory infections in primary care: quasi-experimental trial	Intervention was a Clinical decision support system as a HER progress note template to be available at the point of care. It does not describe a visual analytics tool
[15]	Bourgeois et al., 2010	Impact of a computerized template on antibiotic prescribing for acute respiratory infections in children and adolescents	Intervention was a Clinical decision support system with reminders and visit note template. It does not describe a visual analytics tool
[16]	Diaz et al., 2020	Impact of a Personalized Audit and Feedback Intervention on Antibiotic Prescribing Practices for Outpatient Pediatric Community-Acquired Pneumonia	Intervention arm received monthly feedback via a secure closed-messaging system within the EMR (InBasket messaging) about management of a case of CAP that provider saw the month prior based on manual chart review by 1 of 2 investigators. It does not describe a visual analytics tool
[17]	Moulin et al., 2021	Impact of weekly clinical audits and feedback on consumption of protected anti-gram negative antibiotics in 8 hospitals of the French-speaking part of Switzerland	Conference abstract

n	Authors/year	Title	Reason for exclusion
[18]	Chan et al., 2015	Improvement in the appropriateness of outpatient antibiotics prescription: From the role of pharmacist	Conference abstract
[19]	Juszczyk et al., 2017	Incorporating electronic health records data into efficient trial interventions-cluster randomised trial to reduce antibiotic prescribing in primary care	Conference abstract
[20]	Anyanwu et al., 2020	Investigating the mechanism of impact of the Quality Premium initiative on antibiotic prescribing in primary care practices in England: a study protocol.	Type of study and intervention: This study adopted a natural experiment approach in investigating the mechanism of impact of the 2015–2016 QP target on antibiotic prescribing in primary care practices in England, with the preceding financial year as the control
[21]	Munck et al., 1999	Long-lasting improvement in general practitioners' prescribing of antibiotics by means of medical audit.	It does not describe a visual analytics tool based on EHR, the audits was results of actual guidelines on diagnosis and treatment based on the literature.
[22]	Branch-Elliman et al., 2022	Promoting de-implementation of inappropriate antimicrobial use in cardiac device procedures by expanding audit and feedback: protocol for hybrid III type effectiveness/implementation quasi-experimental study	Recruitment Status: Enrolling by invitation NCT05020418
[23]	Stachel et al., 2017	Promoting judicious antibiotic use: Results of an outpatient-based randomized EMR-generated intervention study	Conference abstract
[24]	Seume et al., 2021	Protocol for an 'efficient design' cluster randomised controlled trial to evaluate a complex intervention to improve antibiotic prescribing for CHIldren presenting to primary care with acute COugh and respiratory tract infection: the CHICO study	Protocol research. Correspondence author commented that they are currently writing up the main findings for the final report and publication.
[25]	Trautner et al., 2018	Protocol to disseminate a hospital-site controlled intervention using	Protocol research. Correspondence author

n	Authors/year	Title	Reason for exclusion
		audit and feedback to implement guidelines concerning inappropriate treatment of asymptomatic bacteriuria.	commented that they have not yet published the main clinical outcomes manuscript.
[26]	Mann et al., 2011	Rationale, design, and implementation protocol of an electronic health record integrated clinical prediction rule (iCPR) randomized trial in primary care.	Intervention was a decision support system, (the CPR tool appeared on the provider's screen during a clinical encounter when the provider entered 1 or more keywords in the fields for chief complaint, diagnosis, or orders). It does not describe a visual analytics tool
[27]	Catho et al., 2018	Study protocol for a multicentre, cluster randomised, superiority trial evaluating the impact of computerised decision support, audit and feedback on antibiotic use: the COMPuterized Antibiotic Stewardship Study (COMPASS).	Intervention was decision support system based on guidelines recommendations (The COMPASS CDSS provides recommendations based on local guidelines to physicians prescribing an antimicrobial treatment). It does not describe a visual analytics tool
[28]	Helou et al., 2020	Study protocol for an international, multicentre stepped-wedge cluster randomised trial to evaluate the impact of a digital antimicrobial stewardship smartphone application	Protocol, Recruitment Status: Not yet recruiting NCT03793946
[29]	Kamradt et al., 2018	Sustainable reduction of antibiotic-induced antimicrobial resistance (ARena) in German ambulatory care: study protocol of a cluster randomised trial	It was a multifaceted intervention program for physicians and patients, each arm included three strategies and one of them was Quality circles with data-based feedback for physicians. It does not describe a visual analytics tool
[30]	Schulz et al., 2013	The Use of Best Practice Alerts with the Development of an Antimicrobial Stewardship Navigator to Promote Antibiotic De-escalation in the Electronic Medical Record	It was a decision support system (alert tool). It does not describe a visual analytics tool

n	Authors/year	Title	Reason for exclusion
[31]	Clegg et al., 2021	Improving antibiotic prescribing for pediatric acute respiratory tract infections: A cluster randomized trial to evaluate individual versus clinic feedback	Intervention was an individual peer comparison feedback to group feedback. It does not describe a visual analytics tool
[32]	Waitman et al., 2011	Adopting real-time surveillance dashboards as a component of an enterprisewide medication safety strategy	It was a decision support system (alert tool).
[33]	Hester et al., 2019	Timely Data for Targeted Quality Improvement Interventions: Use of a Visual Analytics Dashboard for Bronchiolitis	Type of study: case study
[34]	Chang et al., 2022	Changing antibiotic prescribing practices in outpatient primary care settings in China: Study protocol for a health information system-based cluster-randomised crossover controlled trial.	Protocol: ISRCTN13817256 Recruitment status No longer recruiting
[35]	Patel et al., 2020	Developing a clinician facing metric dashboard to foster self development and personal improvement in primary care	Conference abstract
[22]	Branch-Elliman et al., 2022	Promoting de-implementation of inappropriate antimicrobial use in cardiac device procedures by expanding audit and feedback: protocol for hybrid III type effectiveness/implementation quasi-experimental study	Protocol Recruitment Status: Enrolling by invitation
[36]	Melnick et al., 2022	User centered clinical decision support to implement initiation of buprenorphine for opioid use disorder in the emergency department: EMBED pragmatic cluster randomized controlled trial	Condition: it is not focused on antibiotic prescribing
[37]	Pankhurst et al., 2021	Rapid adaptation of a local healthcare digital system to COVID-19: The experience in Birmingham (UK)	Type of study: case study Condition: it is not focused on antibiotic prescribing
[38]	Xie et al., 2022	Effectiveness of clinical dashboards as audit and feedback or clinical decision support tools	Type of study: Systematic review

n	Authors/year	Title	Reason for exclusion
		on medication use and test ordering: a systematic review of randomized controlled trials	
[39]	Pons-Mesquida et al., 2022	Impact of a system to assist in clinical decision-making in primary healthcare in Catalonia: prescription Self Audit	Type of study: cross-sectional study Condition: it is not focused on antibiotic prescribing
[40]	Prasad et al., 2022	Guiding Efficient, Effective, and Patient-Oriented Electrolyte Replacement in Critical Care: An Artificial Intelligence Reinforcement Learning Approach	Type of study: retrospective study Condition: it is not focused on antibiotic prescribing

References

1. Gentry, E.; Kowalkowski, M.; Burns, R.; Sweeney, C.; Collins, C.; Ann Medaris, L.; Spencer, M.; Handy, E.; Davidson, L. A 20/20 Vision: Successful Integration of a Prescribing Dashboard for Outpatient Antimicrobial Stewardship to Target 20% Reduction by the Year 2020. *Open Forum Infect. Dis.* **2019**, *6*, S49–S49, doi:10.1093/ofid/ofz359.109.

2. Gonzales, R.; Anderer, T.; McCulloch, C.E.; Maselli, J.H.; Bloom, F.J.; Graf, T.R.; Stahl, M.; Yefko, M.; Molecavage, J.; Metlay, J.P. A Cluster Randomized Trial of Decision Support Strategies for Reducing Antibiotic Use in Acute Bronchitis. *JAMA Intern. Med.* **2013**, *173*, 267, doi:10.1001/jamainternmed.2013.1589.

3. DiBardino, D.M.; Weiss, C.H.; Sung, N.; Rho, J.; Collander, B.; Wunderink, R.G. An Electronic Checklist Vs. Prompting Physicians To Decrease Empirical Antibiotic Utilization. In Proceedings of the C24. THIS CAN BE DONE BETTER: QUALITY, SAFETY AND PROCESS IMPROVEMENT IN CRITICAL CARE; American Thoracic Society, May 2012; pp. A4012–A4012.

4. Rutten, J.J.S.; van Buul, L.W.; Smalbrugge, M.; Geerlings, S.E.; Gerritsen, D.L.; Natsch, S.; Sloane, P.D.; van der Wouden, J.C.; Twisk, J.W.R.; Hertogh, C.M.P.M. An Electronic Health Record Integrated Decision Tool and Supportive Interventions to Improve Antibiotic Prescribing for Urinary Tract Infections in Nursing Homes: A Cluster Randomized Controlled Trial. *J. Am. Med. Dir. Assoc.* **2022**, *23*, 387–393, doi:10.1016/j.jamda.2021.11.010.

5. Gjelstad, S.; Fetveit, A.; Straand, J.; Dalen, I.; Rognstad, S.; Lindbaek, M. Can Antibiotic Prescriptions in Respiratory Tract Infections Be Improved? A Cluster-Randomized Educational Intervention in General Practice - The Prescription Peer Academic Detailing (Rx-PAD) Study [NCT00272155]. *BMC Health Serv. Res.* **2006**, *6*, 1–12, doi:10.1186/1472-6963-6-75.

6. Gulliford, M.C.; van Staa, T.; McDermott, L.; Dregan, A.; McCann, G.; Ashworth, M.; Charlton, J.; Grieve, A.P.; Little, P.; Moore, M. V; et al. Cluster Randomised Trial in the General Practice Research Database: 1. Electronic Decision Support to Reduce Antibiotic Prescribing in Primary Care (ECRT Study). *Trials* **2011**, *12*, 115.

7. Feldstein, D.A.; Hess, R.; McGinn, T.; Mishuris, R.G.; McCullagh, L.; Smith, P.D.; Flynn, M.; Palmisano, J.; Doros, G.; Mann, D. Design and Implementation of Electronic Health Record Integrated Clinical Prediction Rules (ICPR): A Randomized Trial in Diverse Primary Care Settings. *Implement. Sci.* **2017**, *12*, 1–11, doi:10.1186/s13012-017-0567-y.

8. Palin, V.; Tempest, E.; Mistry, C.; Van Staa, T.P. Developing the Infrastructure to Support the Optimisation of Antibiotic Prescribing Using the Learning Healthcare System to Improve Healthcare Services in the

Provision of Primary Care in England. *BMJ Heal. Care Informatics* **2020**, *27*, 1–5, doi:10.1136/bmjhci-2020-100147.

9. Litvin, C.; Wessell, A.M.; Nemeth, L.; Mainous, A.G.; Ornstein, S.M. Development and Implementation of a Clinical Decision Support System to Promote Judicious Use of Antibiotics in Primary Care. *J. Investig. Med.* **2011**, *59*, 536, doi:10.231/JIM.0b013e31820bab4c LK - http://jq6am9xs3s.search.serialssolutions.com/?sid=EMBASE&issn=10815589&id=doi:10.231%2FJIM.0b013e31820bab4c&atitle=Development+and+implementation+of+a+clinical+decision+support+system+to+promote+judicious+use+of+antibiotics+in+primary+care&stitle=J.+Invest.+Med.&title=Journal+of+Investigative+Medicine&volume=59&issue=2&spage=536&epage=&aulast=Litvin&aufirst=C.&auinit=C.&aufull=Litvin+C.&coden=&isbn=&pages=536-&date=2011&auinit1=C&auinitm=.

10. Linder, J.A.; Schnipper, J.L.; Tsurikova, R.; Yu, T.; Volk, L.A.; Melnikas, A.J.; Palchuk, M.B.; Olsha-Yehiav, M.; Middleton, B. Documentation-Based Clinical Decision Support to Improve Antibiotic Prescribing for Acute Respiratory Infections in Primary Care: A Cluster Randomised Controlled Trial. *Inform. Prim. Care* **2009**, *17*, 231–240, doi:10.14236/jhi.v17i4.742.

11. Gulliford, M.C.; Prevost, A.T.; Charlton, J.; Juszczyk, D.; Soames, J.; Mcdermott, L.; Sultana, K.; Wright, M.; Fox, R.; Hay, A.D.; et al. Effectiveness and Safety of Electronically Delivered Prescribing Feedback and Decision Support on Antibiotic Use for Respiratory Illness in Primary Care: REDUCE Cluster Randomised Trial. *BMJ* **2019**, *364*, doi:10.1136/bmj.l236.

12. Blair, P.S.; Turnbull, S.; Ingram, J.; Redmond, N.; Lucas, P.J.; Cabral, C.; Hollinghurst, S.; Dixon, P.; Peters, T.; Horwood, J.; et al. Feasibility Cluster Randomised Controlled Trial of a Within-Consultation Intervention to Reduce Antibiotic Prescribing for Children Presenting to Primary Care with Acute Respiratory Tract Infection and Cough. *BMJ Open* **2017**, *7*, 1–11, doi:10.1136/bmjopen-2016-014506.

13. Lesprit, P.; Brun-Buisson, C. Hospital Antibiotic Stewardship. *Curr. Opin. Infect. Dis.* **2008**, *21*, 344–349, doi:10.1097/QCO.0b013e3283013959.

14. Mainous, A.G.; Lambourne, C.A.; Nietert, P.J. Impact of a Clinical Decision Support System on Antibiotic Prescribing for Acute Respiratory Infections in Primary Care: Quasi-Experimental Trial. *J. Am. Med. Informatics Assoc.* **2013**, *20*, 317–324, doi:10.1136/amiajnl-2011-000701.

15. Bourgeois, F.C.; Linder, J.; Johnson, S.A.; Co, J.P.T.; Fiskio, J.; Ferris, T.G. Impact of a Computerized Template on Antibiotic Prescribing for Acute Respiratory Infections in Children and Adolescents. *Clin. Pediatr. (Phila).* **2010**, *49*, 976–983, doi:10.1177/0009922810373649.

16. Diaz, M.C.G.; Handy, L.K.; Crutchfield, J.H.; Cadilla, A.; Hossain, J.; Werk, L.N. Impact of a Personalized Audit and Feedback Intervention on Antibiotic Prescribing Practices for Outpatient Pediatric Community-Acquired Pneumonia. *Clin. Pediatr. (Phila).* **2020**, *59*, 988–994, doi:10.1177/0009922820928054.

17. Moulin, E.; Plüss Suard, C.; Bellini, C.; Christin, L.; Chuard, C.; Clerc, O.; Cometta, A.; Erard, V.; Marchetti, O.; Troillet, N.; et al. Impact of Weekly Clinical Audits and Feedback on Consumption of Protected Anti-Gram Negative Antibiotics in 8 Hospitals of the French-Speaking Part of Switzerland. *Antimicrob. Resist. Infect. Control* **2021**, *10*, doi:10.1186/s13756-021-00974-z0 LK - http://jq6am9xs3s.search.serialssolutions.com/?sid=EMBASE&issn=20472994&id=doi:10.1186%2Fs13756-021-00974-z0&atitle=Impact+of+weekly+clinical+audits+and+feedback+on+consumption+of+protected+anti-gram+negative+antibiotics+in+8+hospitals+of+the+French-speaking+part+of+Switzerland&stitle=Antimicrob.+Resist.+Infect.+Control&title=Antimicrobial+Resistance+and+Infection+Control&volume=10&issue=SUPPL+1&spage=&epage=&aulast=Moulin&aufirst=E.&auinit=E.&aufull=Moulin+E.&c.

18. Chan, Y.H.; Leu, W.-J.; Tsai, C.-S.; Huang, S.-Y.; Lin, Y.-M. Improvement in the Appropriateness of Outpatient Antibiotics Prescription: From the Role of Pharmacist. *Pharmacotherapy* **2015**, *35*, e249, doi:10.1002/phar.1659 LK - http://jq6am9xs3s.search.serialssolutions.com/?sid=EMBASE&issn=02770008&id=doi:10.1002%2Fphar.1659&atitle=Improvement+in+the+appropriateness+of+outpatient+antibiotics+prescription%3A+From+the+role+of+pharmacist&stitle=Pharmacotherapy&title=Pharmacotherapy&volume=35&issue=11&spage=e249&epage=&aulast=Chan&aufirst=Yi+Harn&auinit=Y.H.&aufull=Chan+Y.H.&coden=&isbn=&pages=e249-

&date=2015&auinit1=Y&auinitm=H.

19. Juszczyk, D.; Charlton, J.; Gulliford, M.C. Incorporating Electronic Health Records Data into Efficient Trial Interventions-Cluster Randomised Trial to Reduce Antibiotic Prescribing in Primary Care. In Proceedings of the TRIALS; BIOMED CENTRAL LTD 236 GRAYS INN RD, FLOOR 6, LONDON WC1X 8HL, ENGLAND, 2017; Vol. 18.

20. Anyanwu, P.E.; Pouwels, K.; Walker, A.; Moore, M.; Majeed, A.; J Hayhoe, B.W.; Tonkin-Crine, S.; Borek, A.; Hopkins, S.; Mcleod, M.; et al. Investigating the Mechanism of Impact and Differential Effect of the Quality Premium Scheme on Antibiotic Prescribing in England: A Longitudinal Study. *BJGP Open* **2020**, *4*, 1–12, doi:10.3399/bjgpopen20X101052.

21. Munck, A.P.; Gahrn-Hansen, B.; Søgaard, P.; Søgaard, J. Long-Lasting Improvement in General Practitioners' Prescribing of Antibiotics by Means of Medical Audit. *Scand. J. Prim. Health Care* **1999**, *17*, 185–190, doi:10.1080/028134399750002629.

22. Branch-Elliman, W.; Lamkin, R.; Shin, M.; Mull, H.J.; Epshtein, I.; Golenbock, S.; Schweizer, M.L.; Colborn, K.; Rove, J.; Strymish, J.M.; et al. Promoting De-Implementation of Inappropriate Antimicrobial Use in Cardiac Device Procedures by Expanding Audit and Feedback: Protocol for Hybrid III Type Effectiveness/Implementation Quasi-Experimental Study. *Implement. Sci.* **2022**, *17*, 1–12, doi:10.1186/s13012-022-01186-8.

23. Stachel, A.; Szerencsy, A.; Pulgarin, C.; Fucito, N.; Pham, V. Promoting Judicious Antibiotic Use: Results of an Outpatient-Based Randomized EMR-Generated Intervention Study. *Open Forum Infect. Dis.* **2017**, *4*, S273–S274, doi:10.1093/ofid/ofx163.609.

24. Seume, P.; Bevan, S.; Young, G.; Ingram, J.; Clement, C.; Cabral, C.; Lucas, P.J.; Beech, E.; Taylor, J.; Horwood, J.; et al. Protocol for an "efficient Design" Cluster Randomised Controlled Trial to Evaluate a Complex Intervention to Improve Antibiotic Prescribing for CHIldren Presenting to Primary Care with Acute COugh and Respiratory Tract Infection: The CHICO Study. *BMJ Open* **2021**, *11*, 1–9, doi:10.1136/bmjopen-2020-041769.

25. Trautner, B.W.; Prasad, P.; Grigoryan, L.; Hysong, S.J.; Kramer, J.R.; Rajan, S.; Petersen, N.J.; Rosen, T.; Drekonja, D.M.; Graber, C.; et al. Protocol to Disseminate a Hospital-Site Controlled Intervention Using Audit and Feedback to Implement Guidelines Concerning Inappropriate Treatment of Asymptomatic Bacteriuria. *Implement. Sci.* **2018**, *13*, 1–11, doi:10.1186/s13012-018-0709-x.

26. Mann, D.M.; Kannry, J.L.; Edonyabo, D.; Li, A.C.; Arciniega, J.; Stulman, J.; Romero, L.; Wisnivesky, J.; Adler, R.; McGinn, T.G. Rationale, Design, and Implementation Protocol of an Electronic Health Record Integrated Clinical Prediction Rule (ICPR) Randomized Trial in Primary Care. *Implement. Sci.* **2011**, *6*, 109, doi:10.1186/1748-5908-6-109.

27. Catho, G.; De Kraker, M.; Waldispühl Suter, B.; Valotti, R.; Harbarth, S.; Kaiser, L.; Elzi, L.; Meyer, R.; Bernasconi, E.; Huttner, B.D. Study Protocol for a Multicentre, Cluster Randomised, Superiority Trial Evaluating the Impact of Computerised Decision Support, Audit and Feedback on Antibiotic Use: The COMPuterized Antibiotic Stewardship Study (COMPASS). *BMJ Open* **2018**, *8*, 1–9, doi:10.1136/bmjopen-2018-022666.

28. Helou, R.I.; Catho, G.; Peyravi Latif, A.; Mouton, J.; Hulscher, M.; Teerenstra, S.; Conly, J.; Huttner, B.D.; Tängdén, T.; Verbon, A. Study Protocol for an International, Multicentre Stepped-Wedge Cluster Randomised Trial to Evaluate the Impact of a Digital Antimicrobial Stewardship Smartphone Application. *BMJ Open* **2020**, *10*, 1–8, doi:10.1136/bmjopen-2019-033640.

29. Kamradt, M.; Kaufmann-Kolle, P.; Andres, E.; Brand, T.; Klingenberg, A.; Glassen, K.; Poß-Doering, R.; Uhlmann, L.; Hees, K.; Weber, D.; et al. Sustainable Reduction of Antibiotic-Induced Antimicrobial Resistance (ARena) in German Ambulatory Care: Study Protocol of a Cluster Randomised Trial. *Implement. Sci.* **2018**, *13*, 1–10, doi:10.1186/s13012-018-0722-0.

30. Schulz, L.; Osterby, K.; Fox, B. The Use of Best Practice Alerts with the Development of an Antimicrobial Stewardship Navigator to Promote Antibiotic De-Escalation in the Electronic Medical Record. *Infect. Control Hosp. Epidemiol.* **2013**, *34*, 1259–1265, doi:10.1086/673977.

31. Clegg, H.W.; Ezzo, S.J.; Flett, K.B.; Anderson, W.E. Improving Antibiotic Prescribing for Pediatric Acute Respiratory Tract Infections: A Cluster Randomized Trial to Evaluate Individual versus Clinic Feedback. *Antimicrob. Steward. Healthc. Epidemiol.* **2021**, *1*, 1–7, doi:10.1017/ash.2021.212.

32. Waitman, L.R.; Phillips, I.E.; McCoy, A.B.; Danciu, I.; Robert, M.; Halpenny, M.S.; Nelsen, C.L.; Johnson, D.C.; Starmer, J.M.; Peterson, J.F. Adopting Real-Time Surveillance Dashboards as a Component of an Enterprisewide Medication Safety Strategy. *Jt. Comm. J. Qual. Patient Saf.* **2011**, *37*, 326–332, doi:10.1016/s1553-7250(11)37041-9.

33. Hester, G.; Lang, T.; Madsen, L.; Tambyraja, R.; Zenker, P. Timely Data for Targeted Quality Improvement Interventions: Use of a Visual Analytics Dashboard for Bronchiolitis. *Appl. Clin. Inform.* **2019**, *10*, 168–174, doi:10.1055/s-0039-1679868.

34. Chang, Y.; Yao, Y.; Cui, Z.; Yang, G.; Li, D.; Wang, L.; Tang, L. Changing Antibiotic Prescribing Practices in Outpatient Primary Care Settings in China: Study Protocol for a Health Information System-Based Cluster-Randomised Crossover Controlled Trial. *PLoS One* **2022**, *17*, e0259065, doi:10.1371/journal.pone.0259065.

35. Patel, T.; Davis, P.; Faldon, L. 70 Developing a Clinician Facing Metric Dashboard to Foster Self Development and Personal Improvement in Primary Care. In Proceedings of the Abstracts; BMJ Publishing Group Ltd, November 16 2020; Vol. 4, p. A26.2-A26.

36. Melnick, E.R.; Nath, B.; Dziura, J.D.; Casey, M.F.; Jeffery, M.M.; Paek, H.; Soares, W.E.; Hoppe, J.A.; Rajeevan, H.; Li, F.; et al. User Centered Clinical Decision Support to Implement Initiation of Buprenorphine for Opioid Use Disorder in the Emergency Department: EMBED Pragmatic Cluster Randomized Controlled Trial. *BMJ* **2022**, *377*, e069271, doi:10.1136/bmj-2021-069271.

37. Pankhurst, T.; Atia, J.; Evison, F.; Gallier, S.; Lewis, J.M.; McKee, D.; Ryan, S.; Sapey, E.; Ball, S.; Coleman, J.J. Rapid Adaptation of a Local Healthcare Digital System to COVID-19: The Experience in Birmingham (UK). *Heal. Policy Technol.* **2021**, *10*, 100568, doi:10.1016/j.hlpt.2021.100568.

38. Xie, C.X.; Chen, Q.; Hincapié, C.A.; Hofstetter, L.; Maher, C.G.; Machado, G.C. Effectiveness of Clinical Dashboards as Audit and Feedback or Clinical Decision Support Tools on Medication Use and Test Ordering: A Systematic Review of Randomized Controlled Trials. *J. Am. Med. Informatics Assoc.* **2022**, *00*, 1–13, doi:10.1093/jamia/ocac094.

39. Pons-Mesquida, M.À.; Oms-Arias, M.; Figueras, A.; Diogène-Fadini, E. Impact of a System to Assist in Clinical Decision-Making in Primary Healthcare in Catalonia: Prescription Self Audit. *BMC Med. Inform. Decis. Mak.* **2022**, *22*, 1–12, doi:10.1186/s12911-022-01809-6.

40. Prasad, N.; Mandyam, A.; Chivers, C.; Draugelis, M.; Hanson, C.W.; Engelhardt, B.E.; Laudanski, K. Guiding Efficient, Effective, and Patient-Oriented Electrolyte Replacement in Critical Care: An Artificial Intelligence Reinforcement Learning Approach. *J. Pers. Med.* **2022**, *12*, doi:10.3390/jpm12050661.
