# Supplementary material for: The Effectiveness of Interactive Dashboards to Optimise Antibiotic Prescribing in Primary Care: A Systematic Review
Source: Antibiotics (Basel). 2023 Jan 10;12(1):136. doi: 10.3390/antibiotics12010136 (PMC9854857; doi:10.3390/antibiotics12010136)
Supplement: Supplementary file 1 [file antibiotics-12-00136-s001.zip › Table S2 - Characteristics of studies included.pdf]

Table S2. Characteristic of studies included

Study ID	Design of study	n° registration	Country	Sample size	Participants (n)	Intervention	Duration of intervention	Control	Outcomes	Sources of funding	Reported limitations	Key conclusions of the study authors
Du Yan 2021 [1]	A two-arm parallel randomized controlled trial	-	United states	Not report	45 clinicians employed at a single telemedicine company, Doctor On Demand (22= intervention group, 23= control group)	**Education plus individualized prescribing feedback dashboard** Education through two components: a 1-h slides-based presentation on national consensus treatment guidelines for acute respiratory tract infections (ARTIs), and online continuing medical education course + Individualized feedback via an online dashboard which showed each physicians their personal rate of antibiotic prescription and practice-wide prescribing rates for upper respiratory infection (URI), bronchitis, sinusitis, and pharyngitis. Dashboards summarized antibiotic prescription rates for the previous month starting May 2018.	6 months (May 2018 to Nov 2018)	It received education through two components	**Primary outcome**: -AP rates (For each of the four diagnostic categories: URI, bronchitis, sinusitis, and pharyngitis) **Secondary outcomes:** -The proportion of total visits diagnosed as sinusitis or pharyngitis over time	Not reported	-There were changing time trends throughout the study which were not directly captured. -There may be limits in generalizability as the majority of clinicians are family medicine -The fact that clinicians were aware of being observed, or the Hawthorne effect, may have played a role in the global reductions in antibiotic prescriptions. -Data were extracted from an electronic medical record without a separate database, which may have resulted in misclassification bias or incorrect data were entered,	- Individualized prescribing feedback dashboards plus education to telemedicine clinicians was more effective than education alone in reducing antibiotic prescriptions for upper respiratory infections and bronchitis, but not for sinusitis or pharyngitis. -Results suggest education alone may be quite effective, given the reductions in antibiotic use seen in both arms over the study period -Future studies should examine the long-term impact of education and feedback interventions, and maintenance of antibiotic

Study ID	Design of study	n° registration	Country	Sample size	Participants (n)	Intervention	Duration of intervention	Control	Outcomes	Sources of funding	Reported limitations	Key conclusions of the study authors
											and missing data.	prescription reductions.
Daneman 2021 [2,3]	An embedded randomized controlled trial	NCT03807466	Canada	Assuming that standard audit and feedback would achieve an 11% improvement in antibiotic prescribing, it determined that to detect a further 3% incremental improvement with the dynamic report they would require 786, 352, or 174 physicians if standard deviation was 15%, 10%, or 7% (power 0.80, 2-tailed alpha 0.05)	343 physicians (172= intervention group, 171= control group 1)<br><br>895 physicians control group 2: Not Enrolled in Audit and Feedback	**MyPractice report as a novel dynamic, online dashboard**<br>-Both versions of the report were developed with input from infectious diseases, implementation science, information technology, and quality improvement specialists, and improved its design through an iterative, user-centered design process; both included the new antibiotic duration and initiation indicators; both offered the same change ideas; both offered details on data quality and caveats.<br>-Ontario Health launched a voluntary audit and feedback report (MyPractice) in 2015 to provide prescribers with quarterly information on their antipsychotic, benzodiazepine, and other neurotropic medication prescribing in relation to their peers.	12 months<br>Quote: "The 4 quarters of 2019 are the intervention period; the 4 quarters of 2018 serve as the preintervention period."	Group 1: Usual static (PDF) email attachment.<br><br>Group 2: no intervention "Not Enrolled in Audit and Feedback"<br>Both included the new antibiotic duration and initiation indicators; both offered the same change ideas; both offered details on data quality and caveats.<br>*Providing Link or example of visualization tool: Screen shot of a usual report in supplement 3 (see original paper)*	-Antibiotic initiation: proportion of residents initiated on an antibiotic during the quarter<br>-Antibiotic prolonged duration: proportion of antibiotic treatments exceeding 7 days during the quarter	This work was supported by a collaboration across Public Health Ontario, Ontario Health and ICES. ICES is funded by an annual grant from the Ontario Ministry of Health and Long-Term Care (MOHLTC). This study also received funding from: the Canadian Institute for Health Research (CIHR) (grant number 378064 to N. D.).	-It was vulnerable to bias related to voluntary physician participation, but they mitigated this through difference-in-differences analysis that accounted for temporal trends and potential differences in resident characteristics. Furthermore, they applied rigorous, blinded RCT methodology to test a novel feedback strategy.<br>-The intervention period was 1 year, and they have not assessed long-term sustainability. However, lower rates of benzodiazepine and antipsychotic use in the audit and feedback group suggests this	-This population-wide audit and feedback intervention (dynamic + static report) was associated with a reduction in use of prolonged antibiotic treatments but not with measurable reductions in antibiotic initiation.<br>-The small percentage reduction in long duration use by individual prescribers was associated with large reductions in days of antibiotic treatment in the overall.<br>-Audit and feedback is a pragmatic, scalable intervention to improve antibiotic use, and when coupled with evaluation systems using

Study ID	Design of study	n° registration	Country	Sample size	Participants (n)	Intervention	Duration of intervention	Control	Outcomes	Sources of funding	Reported limitations	Key conclusions of the study authors
											intervention will be sustainable, given that those quality indicators were implemented 2 years prior.	administrative databases it could generate sustainable and large reductions in antibiotic use.
Hemkens 2017 [4,5]	Pragmatic randomized trial	NCT01773824	Switzerland	They calculated a necessary sample size of 2900 physicians to detect with a power of 90% (1-β) and a 2-sided significance level of .05 (α) a 5% between-group difference for the primary outcome in the first year. They assumed that physicians who regularly access the online service (expected to be 30% of the interventio	2900 primary care physicians with the highest antibiotic prescription rates in Switzerland (1450=intervention group; 1450=control group)	Physicians received a letter enclosing a quarterly updated single-page graphical overview showing the individual amount of antibiotic prescriptions per 100 consultations in the preceding months and displaying the adjusted average in peer physicians, that is the entire population of Swiss primary care physicians. This letter also included an individual access code to the study website, where we offered more detailed online prescription feedback (for example with details on the prescriptions per age group or sex or for certain antibiotic types) and answers to frequently asked questions on antibiotic use. This mailing also included a response postcard for opting-out of the intervention and evidence-based guidelines for optimized antibiotic use in primary care, adapted for Switzerland. The guidelines focused on the 7 most frequent reasons for antibiotic	2 years (Overall, 8 postal feedbacks were provided, from October 2013 the last in July 2015)	Physicians received no material	**Primary outcome**: The prescribed DDD of any type of antibiotics to any patient per 100 consultations (DDD/100c) in the first year. It was assessed by age groups, sex, and specific antibiotic types They measured the outcomes for the first and second year separately to evaluate early on intervention effects in the first and any weaning effects in the second year.	This study was funded by a grant from the Swiss National Science Foundation (32003B_140997/1) and a grant from the Swiss Academy of Medical Science The Basel Institute for Clinical Epidemiology and Biostatistics was supported in 2013 by an unrestricted grant from Santésuisse, an umbrella association of Swiss social health insurers.	Several issues may explain why they found no association: 1. Switzerland has the lowest antibiotic prescription rates in Europe, thus any strategy to decrease antibiotic use may here be more challenging than elsewhere. 2. They had no individual patient data, only data aggregated by month and physician, and they had no data on diagnoses, hospitalizations, or mortality because such data are not routinely provided by Swiss health	Quarterly personalized prescription feedback over 2 years combined with a 1-time provision of evidence-based guidelines does not reduce antibiotic use. Whether antibiotic use can be reduced in some patient groups like the younger remains to be shown. Given the low costs for implementation, more intense and better tailored prescription feedback approaches merit further evaluation and it should be shown whether they

Study ID	Design of study	n° registration	Country	Sample size	Participants (n)	Intervention	Duration of intervention	Control	Outcomes	Sources of funding	Reported limitations	Key conclusions of the study authors
				n group physicians) would reduce their antibiotic prescriptions by 5%, the remaining physicians by 2% with no change in the control group.		prescribing (AP) in primary care (acute unspecific upper respiratory tract infection, sore throat/acute tonsillitis/pharyngitis, acute rhinosinusitis, acute otitis media, acute bronchitis, community-acquired pneumonia, and uncomplicated urinary tract infection). There was no other change of concomitant care or practice.					care providers to health insurers.<br>3. The time lag between consultation or prescription and database entry decreased the directness of the given feedback in the first year and introduced some noise in the data.<br>4. The aggregated routinely collected claims data do not allow drawing any conclusion on patient-relevant benefits and antibiotic resistance in the community	are associated with patient-relevant benefits and directly impact antibiotic resistance.
Curtis 2021 [6]	Randomized, controlled, parallel-group trial	ISRCTN86418238	England	An illustrative power calculation indicated 80% power to detect a difference of 0.53% on primary prescribing outcome at 95% significance; and	1401 general practices (703= intervention groups (356=group 1, 347=group 2), 698=control group)	Intervention Group 1= The 'Behavioural impact' interventions, optimized for engagement, varied by wave: wave1 tailored broad-spectrum antibiotic feedback, wave2 antibiotic feedback "reminder", with a link to prior evidence of feedback prompting change in AP and an invitation to contact us, wave3 a tailored chart of potential cost savings and more	15 weeks<br>The intervention was sent on three occasions ('waves'), at 5-week intervals.	No intervention<br><br>All practices had access to OpenPrescribing.net usage (Dashboard) for individual practice pages.	**Primary outcome**:<br>-Prescribing outcomes: the difference in proportion of antibiotics which were broad-spectrum, for intervention versus control,	This study was funded by the Health Foundation. The funders had no input into the design or conduct of the study, the analysis or decision to submit	-The national prescribing dataset is considered highly accurate. Page views will include non-participants and be affected by, for example, site updates, social media	A series of simple low-cost tailored written communications had a marginal, but significant increase in information-seeking behaviour among primary care staff. There

Study ID	Design of study	n° registration	Country	Sample size	Participants (n)	Intervention	Duration of intervention	Control	Outcomes	Sources of funding	Reported limitations	Key conclusions of the study authors
				similarly a change of 7.42% on primary engagement outcome, that is, 52 out of 700 interventions leading to a dashboard view		information about other data available at OpenPrescribing.net. Intervention Group 2= The 'Plain' interventions remained consistent by waves "tailored broad-spectrum antibiotic feedback (x3)"			during follow-up. **Secondary outcomes:** -Dashboard engagement outcome: Practices having at least one dashboard view, and Page views per practice		and newsletters; but likely equally affecting both groups. They also measured link access; but some recipients may have used alternative data tools which were not captured.	may have been a small impact on prescribing behaviour. Techniques from 'behavioural insights' did not improve engagement compared with a simple communication but had a greater impact on prescribing.
Linder 2010 [7]	Cluster randomized, controlled clinical trial	-	United states	Assuming a baseline antibiotic prescribing rate for ARIs of 35%, alfa of 0.05, and an intraclass correlation coefficient of 0.10, 1798 visits in each group were required to have 80% power to detect a 7% absolute reduction in the antibiotic prescribing rate, a difference we thought would be	27 general practices (14=intervention group, 13= control group): 296548 visits by 136633 patients to 573 clinicians. It was selected from 4 Community health centers 9 Community-based practices 14 Hospital-based practices	**Acute Respiratory Infection (ARI) Quality Dashboard** -The ARI Quality Dashboard contains views of physicians' AP and billing practices for ARI visits. -The ARI Quality Dashboard includes the proportion of ARI visits at which antibiotics were prescribed; the proportion of individual ARI diagnoses (pneumonia, sinusitis, acute bronchitis) at which antibiotics were prescribed; the proportion of broad spectrum AP; the distribution of ARI visits by evaluation and management billing codes (level 1 through 5); and individual patient visit details, including date of service, antibiotic prescribed, antibiotic class, date of prescription, diagnosis codes, and evaluation and	9 months	Usual care	**Primary outcome:** -AP rate for ARIs **Secondary outcomes:** -AP rate for antibiotic-appropriate diagnoses: they selected the more antibiotic-appropriate diagnoses to mask inappropriate prescribing [8], considered antibiotic-appropriate ARI visits those with an ICD-9-CM code for pneumonia (481-486), streptococcal pharyngitis	This study was supported by grants from the Agency for Healthcare Research and Quality and the National Heart, Lung, and Blood Institute (R01HS015169, K08HS014563, and K08HL072806).	Beyond the possible reasons for the lack of effectiveness of the ARI Quality Dashboard: -To identify ARI visits, they relied on billing codes, which remain in many EHRs the only practical way of identifying visit-based diagnoses. In the as-used analysis, they saw no evidence of diagnosis shifting that would be indicated by an increase in the proportion of	-In a cluster randomized controlled trial, they found the introduction of an EHR-based quality report, the ARI Quality Dashboard, did not result in improved antibiotic prescribing. -Antibiotic prescribing rates, even for non—antibiotic-appropriate diagnoses, were generally high. -EHR-based quality reporting, as part of "meaningful

Study ID	Design of study	n° registration	Country	Sample size	Participants (n)	Intervention	Duration of intervention	Control	Outcomes	Sources of funding	Reported limitations	Key conclusions of the study authors
				clinically significant		management billing codes.<br>-It included billing data to provide a sense of a financial incentive to physicians.<br>-Physicians accessed the ARI Quality Dashboard from the EHR Reports Central area, which contained about 10 other reports about preventive and chronic disease management. A physician could “drill down” to any patient’s medical record directly from the ARI Quality Dashboard to review patient details and export the report for additional follow-up or analysis.<br>-The ARI Quality Dashboard displayed visit and prescribing data for the previous year and was automatically updated monthly.			(034.0), sinusitis (461 and 473), and otitis media (381 and 382).<br>-AP rate for non—antibiotic-inappropriate diagnoses If a patient had multiple ARI diagnoses at a visit, they counted that visit only once, giving preference to more antibiotic-appropriate diagnoses.<br>-ARI Quality Dashboard Use: proportion of intervention physicians who used the ARI Quality Dashboard at least once		antibiotic-appropriate visits and no change in antibiotic prescribing for all ARIs combined.<br>-To measure the outcomes, they relied on EHR antibiotic prescribing, which would miss antibiotic prescribing that occurred in the absence of a visit or that clinicians phoned into a pharmacy and did not enter into the EHR.<br>-Their examination of aggregate oral antibiotic prescribing for ARIs also could have masked important differences in specific antibiotic choice, like a decrease in broad-spectrum antibiotic prescribing.<br>-They conducted this study using an	use,” may not improve care in the absence of other changes to primary care practice.<br>-The meaningful use criteria are supposed to be both “ambitious and achievable,” but each of the meaningful use criteria also should be effective. Perhaps even more than the introduction of most new medical treatments, the meaningful use criteria, because of their broad reach, are major healthcare interventions. Each of the present and forthcoming meaningful use criteria should be rigorously evaluated and shown to be effective in improving quality.

Study ID	Design of study	n° registration	Country	Sample size	Participants (n)	Intervention	Duration of intervention	Control	Outcomes	Sources of funding	Reported limitations	Key conclusions of the study authors
											advanced, homegrown EHR in use for a minimum of 3 years, in academically affiliated primary care practices.	
Shen 2018 [9]	A randomized controlled trial design (cluster)	The study was not registered since randomization was applied only to clinics and not to patients.	China	Not report	12 intervention and 12 control villages clinics in Anhui.<br>These clinics had 65 village doctors.<br>A total of 1048 patients completed the observation and interview, including 532 patients at baseline (intervention=269 out of 284, control= 263 out of 274) and 516 patients at endpoint (intervention=262 out of 272, control=254 out of 265).	**Just-in-Time Information and Feedback (JITIF)**<br>- The information component consisted of a set of theory and evidence-based ingredients, including operation guidelines, public commitment, and takeaway information (patients to take home).<br>- The feedback component of JITIF told each participating doctor about his or her performance scores (PSs) and percentages of prescribed antibiotics use (ABU). The PSs for any individual doctor were based on the records of his or her management of symptomatic infection patients in the past 3 months and were rated by a panel of experts on care of infectious diseases according to a pre-set checklist. The percentages of prescribed ABU were also based on the same records and for the same time period but calculated automatically by the Web-based support system.<br>Any PS (or percentage of ABU) for a given doctor was presented in red, yellow, and green, respectively, if it fell	12 months	That is not clear (Usual care)	**Primary outcome:**<br>- Changes in AP: changes between baseline and endpoint and between control and intervention groups in terms of percentages of patients with symptomatic respiratory tract infections (RTIs) or gastrointestinal tract infections (GTIs) being prescribed with oral, intravenous, and injection antibiotics	Development of the primitive project protocol was supported by the China-UK Prosperity Fund (grant number: PPY CHN 1590/15SS19), whereas implementation of the study by the Science Foundation of China (grant number: 81661138001).	- The observation-induced interferences on the practice behaviours. When being observed, the doctors may be more compliant to authorized guidelines. To minimize such influences, the observation on the control and intervention arms used the same observers and identical protocol. However, doctors on the intervention arm were given detailed references, SOPs, and feedback, and thus they knew much better about what they were	Excessive use of antibiotics was very prevalent, and most essential service procedures for patients with symptomatic infections were not commonly practiced at primary care settings in rural Anhui, China. JITIF was effective in reducing antibiotic use and improving service procedures.

Study ID	Design of study	n° registration	Country	Sample size	Participants (n)	Intervention	Duration of intervention	Control	Outcomes	Sources of funding	Reported limitations	Key conclusions of the study authors
						below (or above), within, and above (or below) the interquartile range of the same PS (or percentage of ABU) for all the participating doctors assessed in the same time period.					expected to do than those in the control group.<br>-Nonblinded data collection: the field data collectors may have given, due to various reasons, more positive ratings to intervention than the control groups since they knew the grouping, though the combination of the data quality control measures may have helped in keeping to a minimum.<br>-The use of antibiotics prescription as the primary measure in assessing JITIF efficacy. Given the prevalent use of antibiotics (as high as over 86% for symptomatic RTI or GTI patients), there are reasons to	

Study ID	Design of study	n° registration	Country	Sample size	Participants (n)	Intervention	Duration of intervention	Control	Outcomes	Sources of funding	Reported limitations	Key conclusions of the study authors
											believe that JITIF helped in reducing excessive antibiotics prescription and thus is beneficial. However, less prescribed antibiotics may not necessarily mean better outcomes for all the patients. And there is still a need of a further outcome-oriented evaluation, for example, linking JITIF with recovery from RTIs or GTIs and direct and indirect costs due to the infections.	
Elouafkaoui 2016 [10,11]	A partial factorial cluster randomised controlled trial	ISRCTN49204710	Scotland	The comparison between the control group (n = 163) and the intervention group (n = 632) had 80% power to detect a 12% decrease in overall antibiotic	795 general dental practices (632= intervention group (158 in each group), 163= control group)<br><br>2566 dentists (intervention group = 567, control group= 1999)	Group 1: Individualised graphical Audit & Feedback (A&F) **without** a written behaviour change message (BCM) and **without** a health board comparator (HB)<br>Group 2: Individualised graphical Audit & Feedback (A&F) **without** BCM and **with** a HB<br>Group 3: Individualised graphical Audit & Feedback (A&F) **with** BCM and **without** HB<br>Group 4: Individualised graphical Audit &	The interval between receiving A&F was varied according to allocation, with A&F received at either 0 and 6 months or at 0, 6 and 9 months	No A&F (current practice)	**Primary outcome:**<br>*AP rates*<br>-All antibiotic items/100 claims<br>-DDD - defined daily dose- (all antibiotics)/100 claims<br>Secondary outcome prescribing rates:	This study was conducted as part of the TRiaDS programme of implementation research which is funded by NHS Education for Scotland (NES). The Health Services Research Unit which is funded by the Chief Scientist Office of the Scottish	-One potential limitation is the relatively short duration of the trial.<br>-The use of routinely collected datasets presents limitations as well as strengths. PRISMS collects dispensing	-This study has successfully demonstrated the potential to fully embed RAPiD-style A&F within routine service delivery. Through its collaborative links with dental healthcare

Study ID	Design of study	n° registration	Country	Sample size	Participants (n)	Intervention	Duration of intervention	Control	Outcomes	Sources of funding	Reported limitations	Key conclusions of the study authors
				prescribing.		Feedback (A&F) **with** BCM and **with** HB<br>A&F<br>-A line graph plotting the individual dentist's monthly AP rate presented on A single side of paper.<br>-The graph was derived using two routinely collected electronic healthcare datasets held centrally by the Information Services Division of NHS National Services Scotland<br>-With or without a health board comparator: the inclusion of an additional line to the A&F graph plotting the monthly AP rate of all dentists in an individual dentist's health board.<br>- Written behaviour change message: to construct this message, guidance recommendations for AP when managing patients with bacterial infections were coded for the presence/absence of behaviour change techniques.			-Amoxicillin 3g/100 claims<br>-DDD (Amoxicillin 3g)/100 claims<br>-Broad spectrum antibiotics/100 claims (clindamycin, co-amoxiclav, clarithromycin, cefalexin, and cefradine)<br>-DDD (Broad spectrum antibiotics)/100 claims	Government Health and Social Care Directorates supported the study. The funder had no influence over the design, conduct, analysis and write up of the study.	rather than prescribing data, and MIDAS is a repository for remuneration data rather than treatment provided. Claims for payment for dental treatment are submitted at the end of a course of treatment. In some instances, a course of treatment may be delivered over a number of weeks, while an antibiotic may be prescribed and dispensed at any time during this period. Thus, only a proxy measure of the monthly rate of antibiotic prescribing could be obtained from these datasets	policymakers, TRiaDS is currently identifying the best way to take this forward. This will provide a mechanism to test and evaluate a range of interventions to further improve dentists' antibiotic prescribing.<br>-The rigorous trial design and the theory-based qualitative process evaluation provide a robust evaluation of A&F in antibiotic prescribing in dental primary care. It has helped elucidate the mechanisms by which A&F works best and has created a platform for further research to adapt and refine the intervention to achieve maximum benefit. This

Study ID	Design of study	n° registration	Country	Sample size	Participants (n)	Intervention	Duration of intervention	Control	Outcomes	Sources of funding	Reported limitations	Key conclusions of the study authors
												study paves the way for applying the methodology in different contexts with different target behaviours, not only in dentistry but in other healthcare settings as well.
Chang 2020 [12]	Cluster randomized; crossover r open controlled trial	ChiCTR1900021823	China	Sample size calculation was based on a 5% difference in antibiotic prescription rate between the intervention group and control group with α = 0.05 and β = 0.2. Two independent means (two-tailed test) was used to calculate the sample size, with 63 physicians per group. Due to the cluster design,	164 Physicians (primary care institutions include township public hospitals or community health service centers in China) 82=intervention group, 82= control group<br><br>It was selected from 31 primary care institutions	The feedback included an individual ranking score (peer comparison), statistic information about the diagnosis and antibiotics<br>-The top five diseases of patients seen by the physician over the previous 10 days are shown in the top left-hand corner of the screen.<br>-The start and stop time for the previous 10 days, as well as the number of prescriptions given during this period and department ranking, are shown in the top right-hand corner of the screen.<br>-Statistics on the antibiotic frequency and prescription rate of each antibiotic prescribed appears in the bottom left-hand corner of the screen. The bottom right corner shows precautions and contraindications for antibiotics being use.<br>-The feedback information would disappear after the physician presses the	3 months<br>The feedback intervention was updated every 10 days.	Not intervention: Physicians assigned to the control group would not be given any such feedback.	**Primary outcome:** Antibiotic prescription rates: the 10-day AP rate of physicians: number of antibiotic prescriptions divided by the total number of prescriptions in each 10-day time period (the term "prescription" as used here in referred to one drug).	The trial was funded by the China Medical Board under the project "A second collaborative program to improve the health research capacity of western medical universities in China and Prince of Songkla University".<br>Data collection was favourably supported by the National Natural Science Foundation of China Grant on "Research on feedback intervention mode of antibiotic prescription control in primary medical institutions based on the	-Although lower antibiotic prescription rates can effectively reduce the risk of antibiotic resistance, it does not mean that all antibiotics are prescribed rationally. In addition, there was no judgment in their system that was related to the appropriateness of antibiotic prescriptions. The judgment of the appropriateness of antibiotics could be included in	Antibiotic prescription rates were clearly reduced by this computer network-based feedback program. Compared with other interventions, this intervention's stable reduction in prescription rates and higher compliance among physicians may be more attractive to township public hospitals of developing countries. At the same time, the risk of antibiotic

Study ID	Design of study	n° registration	Country	Sample size	Participants (n)	Intervention	Duration of intervention	Control	Outcomes	Sources of funding	Reported limitations	Key conclusions of the study authors
				they assumed that physicians from the same hospital (cluster) would be more similar than physicians from another hospital in terms of antibiotic prescription rates. They assumed that intra-class correlation would be about 0.05 on the basis of a previous similar study.		escape button on the keyboard. Thus, the intervention maintains confidentiality for physicians who are also free to read the information or ignore it totally.				depth graph neural network technology" (71964009) and the Guizhou Innovative Talent Foundation (2016-4015).	future studies.<br>-The relatively short study may have missed possible seasonal effects, which can affect antibiotic prescription rates. However, although prescription rates vary depending on the season, this variation should have little impact on the effectiveness of the intervention since comparisons were made at the same point in time. If seasonal effects are important then future studies should be conducted covering a period of at least one year.	resistance in developing countries can be effectively reduced.
Jones 2021 [13]	A controlled before-and-after study (quasi-experimental)	-	United states	It is unclear<br><br>The intervention was performed at these locations	Before=157 (116= intervention group, 41=control group)	Peer comparison with behavioural feedback intervention (Dashboard): This dashboard allowed users to visualize and assess their own inappropriate prescribing rates in relation to those	20 months April 1 2018 to Dec 31, 2019. Attending physicians at intervention sites received	Not intervention	**Primary outcome:**<br>-Overall acute respiratory infections (ARI) prescribing:	This research was supported in part by the Agency for Healthcare Research and Quality (AHRQ), U.S. Department	-Using ICD-10 codes to identify encounters for inclusion, it is possible that they missed	-The inappropriate prescribing rate remained stable in the control group but

Study ID	Design of study	n° registration	Country	Sample size	Participants (n)	Intervention	Duration of intervention	Control	Outcomes	Sources of funding	Reported limitations	Key conclusions of the study authors
				out of convenience	After=159 attending physician (114= intervention group, 45=control group)<br><br>(5 emergency department hospitals)	of their peers (users were blinded to the names of other prescribers).	biannual e-mails with their inappropriate prescribing rate and had access to a dashboard that was updated daily showing their performance relative to their peers.		the proportion of encounters with a diagnosis for an ARI<br>**Secondary outcomes:**<br>- ARI encounters in which antibiotics are inappropriate, no. (% prescribed)<br>-ARI encounters in which antibiotics are or may be appropriate, no. (% prescribed). They followed the Meeker et al [14] methods to develop a list ICD-10 Code for upper respiratory system conditions for which antibiotics were considered inappropriate. The list was reviewed by clinicians practicing in the Johns Hopkins Health	of Health and Human Services (HHS) under award number R18 HS026640-02. The authors are solely responsible for this document's contents, findings, and conclusions, which do not necessarily represent the views of AHRQ. Readers should not interpret any statement in this report as an official position of AHRQ or of HHS.	relevant encounters due to a missing diagnosis code for a respiratory condition that warrants or may warrant antibiotics.<br>-The target and control EDs are located in the same geographic area, they draw from different patient populations, which may bias decision making.<br>-They do not know of anything specific, in the context of national attention to the topic of inappropriate prescribing and antibiotic stewardship, other initiatives to change prescribing may have also been implemented at the hospitals differentially.<br>-The intervention	decreased significantly at the intervention sites between the pre- and post-intervention periods.<br>-These findings suggest that implementation of the intervention was associated with improved antibiotic prescribing for ARIs.<br>-Although other studies have shown that peer comparison can be effective, they have also shown that without continued intervention the impact on prescribing is not sustained.<br>-As behavioral feedback that provides simple comparisons to others becomes a more accepted method for modifying

Study ID	Design of study	n° registration	Country	Sample size	Participants (n)	Intervention	Duration of intervention	Control	Outcomes	Sources of funding	Reported limitations	Key conclusions of the study authors
									System. Because patients can have multiple diagnoses associated with an encounter, they also compiled a list of diagnoses for which the provision of antibiotics are or may be required. They also used evidence-based guidelines for when antibiotics are appropriate		focused only on attending physicians and no other clinicians, and they examined only antibiotic prescribing without adjudicating antimicrobial selection or duration of prescribing for conditions requiring antibiotics.<br>-The presumed mechanism driving changes in prescribing was peer comparison, the fact that attending physicians knew they were being observed on their prescribing habits (Hawthorne effect) may also have contributed to the outcome.	clinicians' practices, further applications of this approach can be explored to reduce inappropriate prescribing, combat the spread of resistance, and improve patient outcomes
Davidson 2022 [15]	Before-and-after interrupted time series	-	United states	Not report	162 ambulatory family medicine, internal medicine, pediatric medicine, and urgent care	They used a stakeholder-centered design intervention process (intervention development, education campaign, dashboard, Education)	(1) a pre-intervention baseline period (April 2016–November 2017) followed by	Not applicable	**Secondary outcomes:** Rates of inappropriat e AP: it was calculated as the number of	The CHOSEN initiative was supported by a 2-year grant from The Duke Endowment. The CHOSEN initiative was	1. They used encounter-level billing data differs from other studies that utilized claims data to	-A multidisciplin ary stakeholder approach utilizing an innovative prescribing

Study ID	Design of study	n° registration	Country	Sample size	Participants (n)	Intervention	Duration of intervention	Control	Outcomes	Sources of funding	Reported limitations	Key conclusions of the study authors
					primary-care practices within Atrium Health	Intervention (CHOSEN program) components included **(1)** an antimicrobial stewardship health education campaign for patients and providers. The education campaign occurred over a 6-month period from November 2017 through March 2018 to introduce the CHOSEN educational tools to all practices in the 4 primary-care service lines: internal medicine, family medicine, urgent care, and pediatric medicine. Targeted education provided at leadership and practice meetings focused on how to incorporate the tools into daily practice. The multimedia campaign for patients included social media, mass media, and printed materials, as well as a website (www.atriumhealth.org/germs) that contained patient-specific resources. **(2)** an interactive, provider-facing reporting dashboard for comparing AP behaviours among providers, practices and organizational groupings. The CHOSEN prescribing dashboard was developed in Microsoft Power BI, a business analytics tool used regularly to track care delivery metrics at Atrium Health. Beginning in March 2018, EHR data were integrated monthly into the dashboard for practice and provider	(2) an implementation wash-in period during which all providers were oriented to CHOSEN educational materials and were trained to use the intervention dashboard to obtain prescribing data (December 2017–March 2018) and (3) an intervention period, post-CHOSEN implementation (April 2018– March 2020)<br>-Bi-monthly stakeholder (Physicians, advanced practice providers, pharmacists, nurses and nurse assistants, practice managers, analytics and research professionals, information services, quality leaders, patients, and		encounters with an antibiotic prescription ordered, compared to the total number of eligible encounters (ie, visits with relevant ICD-10 codes)	supported by a diverse group of stakeholders	examine antibiotic prescribing. As a result, they could not verify that prescriptions were filled, or include prescriptions that occurred outside of a patient encounter.<br>2. The focus ofCHOSEN focus on URI across patient groups differed from prior studies that used a tiered system or HEDIS metrics. Although tiered intervention systems offer categorization across different antibiotic prescribing appropriateness, their stakeholders preferred a simpler structure to promote implementation and provider adoption.<br>3. Their choice to bundle performance	dashboard with targeted patient and provider education successfully decreased inappropriate outpatient antibiotic prescribing in a large ambulatory network.<br>-CHOSEN, using this approach, effectively designed and implemented education resources and tools to meet identified needs among both patients and providers for improved understanding and experiences.<br>-CHOSEN demonstrated significant decreases in inappropriate outpatient antibiotic prescribing for upper respiratory tract infections by nearly 20%.

Study ID	Design of study	n° registration	Country	Sample size	Participants (n)	Intervention	Duration of intervention	Control	Outcomes	Sources of funding	Reported limitations	Key conclusions of the study authors
						reporting; only providers who had 10 or more encounters for an indication were included. Physicians, advanced practice providers, quality and performance improvement coordinators, and primary-care administrators had dashboard access.	senior medical leadership) review of dashboard and education topics				feedback, patient and provider education, and media communications into a single, multimodal program limited their ability to measure the effectiveness of any single intervention. Although they studied both awareness and effects of antibiotic education and communications of providers and patients, they could not separate their impacts. They also could not directly measure prescribing dashboard usage based on frequency of provider access; however, all dashboard data were transparent, and providers were encouraged to review and compare their utilization	

Study ID	Design of study	n° registration	Country	Sample size	Participants (n)	Intervention	Duration of intervention	Control	Outcomes	Sources of funding	Reported limitations	Key conclusions of the study authors
											with other providers.	

References

1. Du Yan, L.; Dean, K.; Park, D.; Thompson, J.; Tong, I.; Liu, C.; Hamdy, R.F. Education vs Clinician Feedback on Antibiotic Prescriptions for Acute Respiratory Infections in Telemedicine: A Randomized Controlled Trial. *J. Gen. Intern. Med.* **2021**, *36*, 305–312, doi:10.1007/s11606-020-06134-0.

2. Daneman, N.; Lee, S.M.; Bai, H.; Bell, C.M.; Bronskill, S.E.; Campitelli, M.A.; Dobell, G.; Fu, L.; Garber, G.; Ivers, N.; et al. Population-Wide Peer Comparison Audit and Feedback to Reduce Antibiotic Initiation and Duration in Long-Term Care Facilities with Embedded Randomized Controlled Trial. *Clin. Infect. Dis.* **2021**, *73*, e1296–e1304, doi:10.1093/cid/ciab256.

3. Laur, C.; Sribaskaran, T.; Simeoni, M.; Desveaux, L.; Daneman, N.; Mulhall, C.; Lam, J.; Ivers, N.M. Improving Antibiotic Initiation and Duration Prescribing among Nursing Home Physicians Using an Audit and Feedback Intervention: A Theory-Informed Qualitative Analysis. *BMJ Open Qual.* **2021**, *10*, 1–9, doi:10.1136/bmjoq-2020-001088.

4. Hemkens, L.G.; Saccilotto, R.; Reyes, S.L.; Glinz, D.; Zumbrunn, T.; Grolimund, O.; Gloy, V.; Raatz, H.; Widmer, A.; Zeller, A.; et al. Personalized Prescription Feedback to Reduce Antibiotic Overuse in Primary Care : Rationale and Design of a Nationwide Pragmatic Randomized Trial. *BMC Infect. Dis.* **2016**, 1–8, doi:10.1186/s12879-016-1739-0.

5. Hemkens, L.G.; Saccilotto, R.; Reyes, S.L.; Glinz, D.; Zumbrunn, T.; Grolimund, O.; Gloy, V.; Raatz, H.; Widmer, A.; Zeller, A.; et al. Personalized Prescription Feedback Using Routinely Collected Data to Reduce Antibiotic Use in Primary Care: A Randomized Clinical Trial. *JAMA Intern. Med.* **2017**, *177*, 176–183, doi:10.1001/jamainternmed.2016.8040.

6. Curtis, H.J.; Bacon, S.; Croker, R.; Walker, A.J.; Perera, R.; Hallsworth, M.; Harper, H.; Mahtani, K.R.; Heneghan, C.; Goldacre, B. Evaluating the Impact of a Very Low-Cost Intervention to Increase Practices' Engagement with Data and Change Prescribing Behaviour: A Randomized Trial in English Primary Care. *Fam. Pract.* **2021**, *38*, 373–380, doi:10.1093/fampra/cmaa128.

7. Linder, J.A.; Schnipper, J.L.; Tsurikova, R.; Yu, D.T.; Volk, L.A.; Melnikas, A.J.; Palchuk, M.B.; Olsha-Yehiav, M.; Middleton, B. Electronic Health Record Feedback to Improve Antibiotic Prescribing for Acute Respiratory Infections. *Am. J. Manag. Care* **2010**, *16*, 311–319.

8. Hueston, W.J. Improving Quality or Shifting Diagnoses?: What Happens When Antibiotic Prescribing Is Reduced for Acute Bronchitis? *Arch. Fam. Med.* **2000**, *9*, 933–935, doi:10.1001/archfami.9.9.933.

9. Shen, X.R.; Lu, M.; Feng, R.; Cheng, J.; Chai, J.; Xie, M.; Dong, X.; Jiang, T.; Wang, D. Web-Based Just-in-Time Information and Feedback on Antibiotic Use for Village Doctors in Rural Anhui, China: Randomized Controlled Trial. *J. Med. Internet Res.* **2018**, *20*, doi:10.2196/jmir.8922.

10. Prior, M.; Elouafkaoui, P.; Elders, A.; Young, L.; Duncan, E.M.; Newlands, R.; Clarkson, J.E.; Ramsay, C.R.; Black, I.; Bonetti, D.; et al. Evaluating an Audit and Feedback Intervention for Reducing Antibiotic Prescribing Behaviour in General Dental Practice (the RAPiD Trial): A Partial Factorial Cluster Randomised Trial Protocol.

Implement. Sci. **2014**, *9*, 1–10, doi:10.1186/1748-5908-9-50.

11. Elouafkaoui, P.; Young, L.; Newlands, R.; Duncan, E.M.; Elders, A.; Clarkson, J.E.; Ramsay, C.R. An Audit and Feedback Intervention for Reducing Antibiotic Prescribing in General Dental Practice: The RAPiD Cluster Randomised Controlled Trial. *PLoS Med.* **2016**, *13*, 1–19, doi:10.1371/journal.pmed.1002115.

12. Chang, Y.; Sangthong, R.; McNeil, E.B.; Tang, L.; Chongsuvivatwong, V. Effect of a Computer Network-Based Feedback Program on Antibiotic Prescription Rates of Primary Care Physicians: A Cluster Randomized Crossover-Controlled Trial. *J. Infect. Public Health* **2020**, *13*, 1297–1303, doi:10.1016/j.jiph.2020.05.027.

13. Jones, G.F.; Fabre, V.; Hinson, J.; Levin, S.; Toerper, M.; Townsend, J.; Cosgrove, S.E.; Saheed, M.; Klein, E.Y. Improving Antimicrobial Prescribing for Upper Respiratory Infections in the Emergency Department: Implementation of Peer Comparison with Behavioral Feedback. *Antimicrob. Steward. Healthc. Epidemiol.* **2021**, *1*, 1–6, doi:10.1017/ash.2021.240.

14. Meeker, D.; Linder, J.A.; Fox, C.R.; Friedberg, M.W.; Persell, S.D.; Goldstein, N.J.; Knight, T.K.; Hay, J.W.; Doctor, J.N. Effect of Behavioral Interventions on Inappropriate Antibiotic Prescribing among Primary Care Practices a Randomized Clinical Trial. *JAMA - J. Am. Med. Assoc.* **2016**, *315*, 562–570, doi:10.1001/jama.2016.0275.

15. Davidson, L.E.; Gentry, E.M.; Priem, J.S.; Kowalkowski, M.; Spencer, M.D. A Multimodal Intervention to Decrease Inappropriate Outpatient Antibiotic Prescribing for Upper Respiratory Tract Infections in a Large Integrated Healthcare System. *Infect. Control Hosp. Epidemiol.* **2022**, 1–8, doi:10.1017/ice.2022.83.
