# Supplementary material for: The Effectiveness of Interactive Dashboards to Optimise Antibiotic Prescribing in Primary Care: A Systematic Review
Source: Antibiotics (Basel). 2023 Jan 10;12(1):136. doi: 10.3390/antibiotics12010136 (PMC9854857; doi:10.3390/antibiotics12010136)
Supplement: Supplementary file 1 [file antibiotics-12-00136-s001.zip › Table S3 - Result of outcomes.pdf]

Table S3. Result of outcomes

Study ID	Intervention vs control	Outcome definition	Baseline data in Intervention group	Baseline data in control group	Measure of follow-up in Intervention group	Measure of follow-up in control group	Outcome Measure of result (changes from)			No. of missing participants
							Intervention vs control (post-intervention)	Baseline to intervention group	Baseline to control group	
**Primary outcome**										
Du Yan 2021 [1]	Education plus individualized prescribing feedback dashboard vs Education	Antibiotic prescription rate (APR) -upper respiratory infection (URI)<br>Given the difference in baseline prescription rates, they used an interaction term between group and time period (post versus pre<br>This interaction term is the ratio of two odds ratios (ORs)	15%	18.4%	7.8%	12.8%	OR 0.60 (CI 95% 0.47 to 0.77)	OR 0.49 (CI 95% 0.32 to 0.73)	OR 0.81 (CI 95% 0.69 to 0.95)	Not missing data
		APR - Bronchitis	64%	46.8%	32.1%	35.3%	OR 0.42 (CI 95% 0.32 to 0.55)	OR 0.25 (CI 95% 0.15 to 0.40)	OR 0.60 (CI 95% 0.49 to 0.73)	
		APR - Sinusitis	87.2%	84.1%	76.8%	76.7%	OR 1.05 (CI 95% 0.91 to 1.21)	OR 0.64 (CI 95% 0.51 to 0.81)	OR 0.61 (CI 95% 0.56 to 0.67)	
		APR - Pharyngitis	74.9%	81.3%	65.5%	75.3%	OR 0.91 (CI 95% 0.76 to 1.09)	OR 0.66 (CI 95% 0.48 to 0.90)	OR 0.73 (CI 95% 0.64 to 0.83)	
Hemkens 2017 [2,3]	Personalized prescription feedback vs no intervention (usual care)	**Prescriptions per year (defined daily doses DDD/100c, all antibiotic types), all patients and year 1**	Median 101 (IQR 83 to 130)	Median 101 (IQR 83 to 133)	Median 90.5 (IQR 71.2 to 119.1)	Median 90.3 (IQR 71.8 to 121.2)	Change from Baseline (between-group difference): 0.81% (CI -2.56% to 4.30%)			When they had no prescribing information for a certain

Study ID	Intervention vs control	Outcome definition	Baseline data in Intervention group	Baseline data in control group	Measure of follow-up in Intervention group	Measure of follow-up in control group	Outcome Measure of result (changes from)			No. of missing participants
							Intervention vs control (post-intervention)	Baseline to intervention group	Baseline to control group	
		Prescriptions per year, ≤ 5 years and year 1	Median 70 (IQR 15 to 160)	Median 71 (IQR 0 to 150)	Median 89.5 (IQR 47.7 to 166.9)	Median 82.9 (IQR 48.6 to 164.7)	Change from Baseline (between-group difference): 1.53% (-9.39% to 13.77%)			month they assumed that a physician actually
		Prescriptions per year, 6 - 18 years and year 1	Median 109 (IQR 67 to 168)	Median 107 (IQR 63 to 173)	Median 97.2 (IQR 60.4 to 156.9)	Median 105 (IQR 65.8 to 164.2)	Change from Baseline (between-group difference): -8.61% (-14.87% to -1.90%)			prescribed no antibiotics whenever there was at
		Prescriptions per year, 19 - 65 years and year 1	Median 107 (IQR 85 to 140)	Median 108 (IQR 85 to 144)	Median 94.4 (IQR 73.1 to 126.9)	Median 95.3 (IQR 71.5 to 129.2)	Change from Baseline (between-group difference): -0.17% (-3.58% to 3.36%)			least 1 consultation in this month, otherwise
		Prescriptions per year, > 65 years and year 1	Median 90 (IQR 71 to 122)	Median 91 (IQR 71 to 120)	Median 81.4 (IQR 62.1 to 113.2)	Median 80.3 (IQR 60.3 to 113.7)	Change from Baseline (between-group difference): 1.13% (-2.75% to 5.16%)			they kept the value of antibiotics as missing. They
		Prescriptions per year, Penicillins (beta-Lactams) and year 1	Median 41 (IQR 30 to 56)	Median 40 (IQR 29 to 57)	Median 37.6 (IQR 26.5 to 50.6)	Median 36.6 (IQR 25.9 to 50.7)	Change from Baseline (between-group difference): 1.42% (-2.65% to 5.65%)			did not impute any missing data. In some
		Prescriptions per year, Other beta-Lactams and year 1	Median 7 (IQR 2 to 16)	Median 6 (IQR 2 to 18)	Median 6.6 (IQR 2.3 to 15.8)	Median 6.9 (IQR 2.4 to 16.5)	Change from Baseline (between-group difference): -0.04% (-7.80% to 8.37%)			cases, there was no information on baseline
		Prescriptions per year, Quinolones and year 1	Median 18 (IQR 12 to 26)	Median 17 (IQR 12 to 25)	Median 16.3 (IQR 10.9 to 23.9)	Median 15.5 (IQR 10.2 to 23)	Change from Baseline (between-group difference): -0.82% (-5.42% to 4.00%)			characteristics used for the linear model; then they
		Prescriptions per year, Macrolides, Lincosamides, StGr and year 1	Median 14 (IQR 9 to 23)	Median 15 (IQR 8 to 25)	Median 11.8 (IQR 6.8 to 19.9)	Median 12.4 (IQR 7 to 21)	Change from Baseline (between-group difference): -1.39% (-6.58% to 4.07%)			excluded those physicians

Study ID	Intervention vs control	Outcome definition	Baseline data in Intervention group	Baseline data in control group	Measure of follow-up in Intervention group	Measure of follow-up in control group	Outcome Measure of result (changes from)			No. of missing participants
							Intervention vs control (post-intervention)	Baseline to intervention group	Baseline to control group	
		Prescriptions per year, Tetracyclines and year 1	Median 4 (IQR 1 to 9)	Median 4 (IQR 1 to 9)	Median 4.9 (IQR 2.5 to 9.6)	Median 5.1 (IQR 2.3 to 9.6)	Change from Baseline (between-group difference): 4.77% (-3.78% to 14.08%)			from this analysis.<br>211 Discontinued intervention (opt-out)<br>0 Discontinued control (opt-out)<br>*Analyzed*<br>-Intervention group):<br>44 Excluded from analysis (26 Deregistered from health care system, 7 Data error, 11 Implausible baseline covariates).<br>-Control group: 42 Excluded from analysis, (20 Deregistered from
		Prescriptions per year, Sulfonamides/Trimethoprim and year 1	Median 2 (IQR 1 to 5)	Median 2 (IQR 1 to 5)	Median 2.1 (IQR 0.9 to 4.4)	Median 2 (IQR 0.9 to 4.5)	Change from Baseline (between-group difference): 5.68% (-4.29% to 16.70%)			
		Prescriptions per year, Aminoglycosides and year 1	Median 0 (IQR 0 to 0)	Median 0 (IQR 0 to 0)	Median 0.09 (IQR 0.02 to 0.9)	Median 3.2 (IQR 0.6 to 4.8)	Change from Baseline (between-group difference): 12.89% (-64.10% to 255.0%)			
		**Prescriptions per year (DDD/100c, all antibiotic types), all patients and year 2**	NA	NA	Median 91 (IQR 70.3 to 121.7)	Median 92.7 (IQR 71.5 to 128.3)	Change from Baseline (between-group difference): -1.73% (-5.07% to 1.72%)			
		Prescriptions per year, ≤ 5 years and year 2	NA	NA	Median 80 (IQR 46.7 to 156.1)	Median 84.4 (IQR 46.4 to 160)	Change from Baseline (between-group difference): -2.03% (-13.11% to 10.45%)			
		Prescriptions per year, 6 - 18 years and year 2	NA	NA	Median 101.5 (IQR 63.3 to 163.7)	Median 105.9 (IQR 63.9 to 172.1)	Change from Baseline (between-group difference): -4.10% (-10.78% to 3.07%)			
		Prescriptions per year, 19 - 65 years and year 2	NA	NA	Median 92.8 (IQR 68.8 to 128)	Median 97.7 (IQR 70.9 to 138)	Change from Baseline (between-group difference): -4.59% (-7.91% to -1.16%)			
		Prescriptions per year, > 65 years and year 2	NA	NA	Median 83.7 (IQR 61.4 to 118.5)	Median 83.6 (IQR 61.2 to 122.4)	Change from Baseline (between-group difference): -2.53% (-6.33% to 1.42%)			

**Study ID**	**Intervention vs control**	**Outcome definition**	**Baseline data in Intervention group**	**Baseline data in control group**	**Measure of follow-up in Interventio n group**	**Measure of follow-up in control group**	**Outcome Measure of result (changes from)**			**No. of missing participants**
							**Intervention vs control (post-intervention)**	**Baseline to interventio n group**	**Baseline to control group**	
		Prescriptions per year, Penicillins (beta-Lactams) and year 2	NA	NA	Median 37.8 (IQR 26.7 to 53.8)	Median 38.8 (IQR 26.5 to 56.9)	Change from Baseline (between-group difference): -1.98% (-5.97% to 2.18%)			health care system, 7 Data error, 15 Implausible baseline covariates)
		Prescriptions per year, Other beta-Lactams and year 2	NA	NA	Median 6.2 (IQR 2.2 to 16.1)	Median 7.2 (IQR 2.3 to 17.9)	Change from Baseline (between-group difference): -3.55% (-11.15% to 4.70%)			
		Prescriptions per year, Quinolones and year 2	NA	NA	Median 15.1 (IQR 9.6 to 23.3)	Median 14.3 (IQR 9.4 to 22.4)	Change from Baseline (between-group difference): -1.00% (-5.67% to 3.91%)			
		Prescriptions per year, Macrolides, Lincosamides, StGr and year 2	NA	NA	Median 12.5 (IQR 7 to 21.4)	Median 13.6 (IQR 7.5 to 23.3)	Change from Baseline (between-group difference): -5.71% (-10.75% to -0.38%)			
		Prescriptions per year, Tetracyclines and year 2	NA	NA	Median 5.1 (IQR 2.5 to 9.5)	Median 4.9 (IQR 2.5 to 9.5)	Change from Baseline (between-group difference): 2.81% (-5.91% to 12.34%)			
		Prescriptions per year, Sulfonamides/Trimethopri m and year 2	NA	NA	Median 2 (IQR 0.8 to 4.3)	Median 2.1 (IQR 0.8 to 4.5)	Change from Baseline (between-group difference): 4.94% (-5.42% to 16.44%)			
		Prescriptions per year, Aminoglycosides and year 2	NA	NA	Median 0.2 (IQR 0.05 to 3.1)	Median 3.5 (IQR 1.3 to 9)	Change from Baseline (between-group difference): -43.59% (-84.51% to 105.4%)			
Curtis 2021 [4]	Intervention group 1 + group 2 (The 'Plain' + The 'Behavioural	The difference in proportion of antibiotics which were broad-spectrum, for intervention	12.67%	12.71%	11.25%	11.59%	Regression model indicated this was not	1.42%	1.12%	Not missing data

Study ID	Intervention vs control	Outcome definition	Baseline data in Intervention group	Baseline data in control group	Measure of follow-up in Intervention group	Measure of follow-up in control group	Outcome Measure of result (changes from)			No. of missing participants
							Intervention vs control (post-intervention)	Baseline to intervention group	Baseline to control group	
	impact') vs No intervention	versus control, during follow-up					statistically significant at P < 0.05 (coefficient −0.31%, CI: −0.7% to 0.1%, P = 0.104)			
	Intervention group 1 (Behavioural impact intervention) vs Intervention group 2 (Plain intervention)		12.7%	12.64%	11.07%	11.44%	This difference was significant at P < 0.05 (coefficient 0.41%, CI: 0.007% to 0.8%) P = 0.046)	1.63%	1.2%	
Linder 2010 [5]	Acute Respiratory Infection (ARI) Quality Dashboard vs Usual care	Antibiotic prescribing rate for ARIs: difference in antibiotic prescribing between control and intervention practices for ARI visits in aggregate, adjusted for clustering by clinic	Not reported	Not reported	47%	47%	OR 0.97 (CI 95% 0.7 to 1.4)	-	-	Not missing data (intent-to-intervene analysis)
		Antibiotic prescribing rate for ARIs - ARI Quality Dashboard Users (intervention clinicians who used the ARI Quality	Not reported	Not reported	42%	50%	OR 0.72 (CI 95% 0.54 to 0.96)	-	-	

Study ID	Intervention vs control	Outcome definition	Baseline data in Intervention group	Baseline data in control group	Measure of follow-up in Intervention group	Measure of follow-up in control group	Outcome Measure of result (changes from)			No. of missing participants
							Intervention vs control (post-intervention)	Baseline to intervention group	Baseline to control group	
		Dashboard at least once with intervention clinicians who did not use the ARI Quality Dashboard, adjusted for clustering by clinician)								
Shen XR 2018 [6]	Just-in-Time Information and Feedback vs usual care	Percentages of patients with respiratory tract infections (RTIs) Oral	65.6%	66%	42.3%	60.5%	OR 0.48 (CI 95% 0.31 to 0.73) *	-	-	5% of patients in intervention group – baseline 4% of patients in intervention group – baseline 3% of patients in intervention group – endpoint 4% of patients in intervention group – endpoint
		For RTIs Intravenous	54%	53%	38.4%	51.2%	OR 0.59 (CI 95% 0.39 to 0.91) *	-	-	
		For RTIs any	78.1%	90.3%	64.3%	89.7%	OR 0.21 (CI 95% 0.12 to 0.36) *	-	-	
		For gastrointestinal tract infections (GTIs) Oral	64%	68%	12.8%	62.5%	OR 0.09 (CI 95% 0.03 to 0.26) *	-	-	
		For GTIs Intravenous	58.6%	60%	36.4%	60%	OR 0.38 (CI 95% 0.15 to 0.95) *	-	-	
		For GTIs any	94.7%	96.2%	52.4%	90%	OR 0.40 (CI 95% 0.17 to 0.95) *	-	-	
		For RTIs and/or GTIs Oral	65.3%	66.5%	36.7%	60.9%	OR 0.37 (CI 95% 0.25 to 0.55) *	-	-	
		For RTIs and/or GTIs Intravenous	55.1%	54.6%	38%	52.7%	OR 0.55 (CI 95% 0.37 to 0.81) *	-	-	

Study ID	Intervention vs control	Outcome definition	Baseline data in Intervention group	Baseline data in control group	Measure of follow-up in Intervention group	Measure of follow-up in control group	Outcome Measure of result (changes from)			No. of missing participants
							Intervention vs control (post-intervention)	Baseline to intervention group	Baseline to control group	
		For RTIs and/or GTIs any	88.8%	91.7%	62.3%	89.7%	OR 0.19 (CI 95% 0.11 to 0.32) *	-	-	
Elouafkaoui 2016 [7,8]	All interventions (BCM, written behaviour change message; HB, health board; A&F, audit and feedback) versus Control (No A&F)	All antibiotic items/100 claims	Mean rate:8.5 (SD:9.5)	Mean rate:8.3 (SD:7.2)	Mean rate:7.5 (SD:NR)	Mean rate:7.9 (SD:NR)	Change from baseline (All percentages standardised using control group baseline mean prescribing rate (8.3)) = -5.7% (CI 95% -10.2% to -1.1%)			Intervention= 4%<br>Control = 7%
		DDD (all antibiotics)/100 claims	Mean rate:40.7 (SD:NR)	Mean rate:39.5 (SD:NR)	Mean rate:37.3 (SD:NR)	Mean rate:39.7 (SD:NR)	Change from baseline (Percentages standardised using control group mean prescribing rate (39.5) = -6.6% (-12.5% to -0.7%)			
		Amoxicillin 3g/100 claims	Mean rate:0.1 (SD:NR)	Mean rate:0.1 (SD:NR)	Mean rate:0.1 (SD:NR)	Mean rate:0.1 (SD:NR)	Change from baseline (All percentages standardised using 0.077) = -26.0% (-64.9% to 13.0%)			
		DDD (Amoxicillin 3g)/100 claims	Mean rate:0.5 (SD:NR)	Mean rate:0.4 (SD:NR)	Mean rate:0.4 (SD:NR)	Mean rate:0.5 (SD:NR)	Change from baseline (All percentages standardised using 0.44) = -31.8% (-79.5% to 13.6%)			
		Broad spectrum antibiotics/100 claims (clindamycin, co-amoxiclav, clarithromycin, cefalexin, and cefradine)	Mean rate:0.1 (SD:NR)	Mean rate:0.2 (SD:NR)	Mean rate:0.1 (SD:NR)	Mean rate:0.1 (SD:NR)	Change from baseline (All percentages standardised using 0.15) = -33.3% (-80.0% to 20.0%)			
		DDD (Broad spectrum antibiotics)/100 claims	Mean rate:0.4 (SD:NR)	Mean rate:0.8 (SD:NR)	Mean rate:0.3 (SD:NR)	Mean rate:0.8 (SD:NR)	Change from baseline (All percentages standardised using 0.81) = -33.3% (-79.0% to 12.3%)			
	BCM intervention and A&F versus no BCM intervention and A&F	All antibiotic items/100 claims	Mean rate:8.5 (SD:NR)	Mean rate:8.5 (SD:NR)	Mean rate:7.2 (SD:NR)	Mean rate:7.7 (SD:NR)	Change from baseline (All percentages standardised using control group baseline mean prescribing rate (8.3)) = -6.1% (-10.4% to -1.9%)			Intervention= 4%<br>Control = 7%

Study ID	Intervention vs control	Outcome definition	Baseline data in Intervention group	Baseline data in control group	Measure of follow-up in Intervention group	Measure of follow-up in control group	Outcome Measure of result (changes from)			No. of missing participants
							Intervention vs control (post-intervention)	Baseline to intervention group	Baseline to control group	
		DDD (all antibiotics)/100 claims	Mean rate:41.7 (SD:NR)	Mean rate:39.7 (SD:NR)	Mean rate:36.7 (SD:NR)	Mean rate:38 (SD:NR)	Change from baseline (Percentages standardised using control group mean prescribing rate (39.5) = -5.7% (-10.7% to -0.7%)			
		Amoxicillin 3g/100 claims	Mean rate:0.1 (SD:NR)	Mean rate:0.1 (SD:NR)	Mean rate:0.1 (SD:NR)	Mean rate:0.1 (SD:NR)	Change from baseline (All percentages standardised using 0.077) = -13.0% (-39.0% to 13.0%)			
		DDD (Amoxicillin 3g)/100 claims	Mean rate:0.6 (SD:NR)	Mean rate:0.5 (SD:NR)	Mean rate:0.4 (SD:NR)	Mean rate:0.3 (SD:NR)	Change from baseline (All percentages standardised using 0.44) = -9.1% (-40.9% to 25.0%)			
		Broad spectrum antibiotics/100 claims	Mean rate:0.1 (SD:NR)	Mean rate:0.1 (SD:NR)	Mean rate:0.1 (SD:NR)	Mean rate:0.1 (SD:NR)	Change from baseline (All percentages standardised using 0.15) = -6.7% (-20.0% to 13.3%)			
		DDD (Broad spectrum antibiotics)/100 claims	Mean rate:0.3 (SD:NR)	Mean rate:0.4 (SD:NR)	Mean rate:0.2 (SD:NR)	Mean rate:0.3 (SD:NR)	Change from baseline (All percentages standardised using 0.81) = -6.2% (-21.0% to 8.6%)			
	HB comparator and A&F versus No HB comparator and A&F	All antibiotic items/100 claims	Mean rate:8.6 (SD:NR)	Mean rate:8.4 (SD:NR)	Mean rate:7.4 (SD:NR)	Mean rate:7.5 (SD:NR)	Change from baseline (All percentages standardised using control group baseline mean prescribing rate (8.3)) = -4.3% (-8.6% to 0.1%)			Intervention= 4%<br>Control = 7%
		DDD (all antibiotics)/100 claims	Mean rate:40 (SD:NR)	Mean rate:41.4 (SD:NR)	Mean rate:37.4 (SD:NR)	Mean rate:37.2 (SD:NR)	Change from baseline (Percentages standardised using control group mean prescribing rate (39.5) = -4.2% (-9.4% to 1.1%)			
		Amoxicillin 3g/100 claims	Mean rate:0.1 (SD:NR)	Mean rate:0.1 (SD:NR)	Mean rate:0.1 (SD:NR)	Mean rate:0.1 (SD:NR)	Change from baseline (All percentages standardised using 0.077) = -13.0% (-39.0% to 26.0%)			
		DDD (Amoxicillin 3g)/100 claims	Mean rate:0.5 (SD:NR)	Mean rate:0.6 (SD:NR)	Mean rate:0.3 (SD:NR)	Mean rate:0.4 (SD:NR)	Change from baseline (All percentages standardised using 0.44) = -9.1% (-43.2% to 22.7%)			

Study ID	Intervention vs control	Outcome definition	Baseline data in Intervention group	Baseline data in control group	Measure of follow-up in Interventio n group	Measure of follow-up in control group	Outcome Measure of result (changes from)			No. of missing participants
							Intervention vs control (post-intervention)	Baseline to interventio n group	Baseline to control group	
		Broad spectrum antibiotics/100 claims	Mean rate:0.1 (SD:NR)	Mean rate:0.1 (SD:NR)	Mean rate:0.1 (SD:NR)	Mean rate:0.1 (SD:NR)	Change from baseline (All percentages standardised using 0.15) = 2.0% (-13.3% to 20.0%)			
		DDD (Broad spectrum antibiotics)/100 claims	Mean rate:0.4 (SD:NR)	Mean rate:0.3 (SD:NR)	Mean rate:0.2 (SD:NR)	Mean rate:0.3 (SD:NR)	Change from baseline (All percentages standardised using 0.81) = 4.9% (-11.1% to 19.8%)			
Chang 2020 [9]	The feedback included an individual ranking score (group 1) crossover after 3 months to no intervention (group 2) crossover after 3 months to feedback	The antibiotic prescription rates: the 10-day antibiotic prescription rate of physicians	Mean rate:30.8 (SD:15.5)	Mean rate:35.2 (SD:14.9)	Group 1 in crossover point: mean rate 20.3% end point: mean rate 15.7%	Group 2 in crossover point: mean rate 33.1% end point: mean rate 18.9%	Change from Baseline in period 1 and period 2 (crossover): At the end of the 6-month study period, there was no significant difference in the rate of antibiotic prescriptions between the two groups (P-value = 0.078).	Period 1: the relative decline in antibiotic prescription rate (ΔAPR) among the intervention group was 10.5% (P-value < 0.001) but more pronounced than for the control group, which also exhibited a significant decline in prescription rate (ΔAPR = 2.1%, P-	Period 2: there were significant changes in antibiotic prescription rates for the interventio n group (ΔAPR = 14.2%, P-value < 0.001), as well as for the control group (ΔAPR = 4.6%, P-value < 0.001), and as before, the rate of decline was more	One physician from group 2 was excluded due to IT technical problem, data could not be retrieved

Study ID	Intervention vs control	Outcome definition	Baseline data in Intervention group	Baseline data in control group	Measure of follow-up in Interventio n group	Measure of follow-up in control group	Outcome Measure of result (changes from)			No. of missing participants
							Intervention vs control (post-intervention)	Baseline to interventio n group	Baseline to control group	
								value = 0.002).	pronounce d in the interventio n group	
Jones 2021 [10]	Peer comparison with behavioural feedback intervention vs Not intervention	Overall acute respiratory infections (ARI) prescribing rates: the proportion of encounters with a diagnosis for an ARI	35.9%	38.1%	30.6%	40.6%	-	-	-	Not missing data
**Secondary outcome**										
Du Yan 2021 [1]	Education plus individualized feedback vs Education	Proportion of total visits diagnosed as sinusitis or pharyngitis over time	Not reported	Not reported	Not reported	Not reported	OR 1.36 (CI 95% 1.29 to 1.44)	Not reported	Not reported	Not missing data
Daneman 2021 [11,12]	MyPractice report as a novel dynamic, online dashboard vs Usual static (PDF) email attachment	Antibiotic duration: proportion of antibiotic treatments exceeding 7 days during the quarter	Median 16% (IQR: 7 to 25)	Median 17% (IQR: 8 to 29)	Median 30.1%	Median 31.1%	OR 1.94 (CI 95% 0.88 to 1.23)	Not reported	Not reported	Not missing data (intention to treat analysis)
		Antibiotic initiation: proportion of residents initiated on an antibiotic during the quarter	Median 23% (IQR: 17 to 27)	Median 24% (IQR: 17 to 29)	Median 23.2%	Median 23.8%	OR 0.98 (CI 95% 0.89 to 1.06)	-	-	Not missing data (intention to treat analysis)
Curtis 2021 [4]	Intervention group 1 + group 2 (The 'Plain' + The 'Behavioural impact') vs No intervention (All practices had	Dashboard engagement outcome: Practices having at least one dashboard view	60.9%	56.4%	65.7%	55.9%	Difference in proportion 9.8% (CI 95% 4.76% to 14.9%)	+34 practices (+4.8%)	-4 practices	Not missing data

Study ID	Intervention vs control	Outcome definition	Baseline data in Intervention group	Baseline data in control group	Measure of follow-up in Intervention group	Measure of follow-up in control group	Outcome Measure of result (changes from)			No. of missing participants
							Intervention vs control (post-intervention)	Baseline to intervention group	Baseline to control group	
	access to OpenPrescribing.net usage (Dashboard) for individual practice pages.)									
	Intervention group 1 (Behavioural impact intervention) vs Intervention group 2 (Plain intervention)		61.2%	60.5%	62.4%	69.1%	Difference in proportion 6.8% (CI 95% −0.19% to 13.8%)	+4 practices	+30 practices	
	Intervention group 1 + group 2 (The 'Plain' + The 'Behavioural impact') vs No intervention (All practices had access to OpenPrescribing.net usage (Dashboard) for individual practice pages.)	Dashboard engagement outcome: Page views per practice	Mean: 1.51 (SD: 1.99)	Mean: 1.44 (SD: 2.09)	Mean: 1.75 (SD: 2.12)	Mean: 1.42 (SD: 2.11)	-	Difference of means: +0.24	Difference of means: -0.02	
	Intervention group 1 (Behavioural impact intervention) vs Intervention group		Mean: 1.4 (SD: 1.78)	Mean: 1.63 (SD: 2.18)	Mean: 1.66 (SD: 2.12)	Mean: 1.84 (SD: 2.12)	-	Difference of means: +0.24	Difference of means: +0.24	

Study ID	Intervention vs control	Outcome definition	Baseline data in Intervention group	Baseline data in control group	Measure of follow-up in Intervention group	Measure of follow-up in control group	Outcome Measure of result (changes from)			No. of missing participants
							Intervention vs control (post-intervention)	Baseline to intervention group	Baseline to control group	
	2 (Plain intervention)									
Linder 2010 (5)	Acute Respiratory Infection (ARI) Quality Dashboard vs Usual care	*Antibiotic-appropriate diagnosis:* Subtotal	Not reported	Not reported	65%	64%	Not reported	Not reported	Not reported	Not missing data
		Pneumonia	Not reported	Not reported	41%	37%	Not reported	Not reported	Not reported	
		Streptococcal pharyngitis	Not reported	Not reported	76%	77%	Not reported	Not reported	Not reported	
		Sinusitis	Not reported	Not reported	73%	72%	Not reported	Not reported	Not reported	
		Otitis media	Not reported	Not reported	66%	67%	Not reported	Not reported	Not reported	
		Non–antibiotic appropriate diagnosis: Subtotal	Not reported	Not reported	38%	43%	Not reported	Not reported	Not reported	Not missing data
		Pneumonia	Not reported	Not reported	36%	22%	Not reported	Not reported	Not reported	
		Streptococcal pharyngitis	Not reported	Not reported	43%	62%	Not reported	Not reported	Not reported	
		Sinusitis	Not reported	Not reported	69%	23%	Not reported	Not reported	Not reported	
		Otitis media	Not reported	Not reported	27%	43%	Not reported	Not reported	Not reported	
	Acute Respiratory Infection (ARI) Quality Dashboard (only intervention group)	ARI Quality Dashboard Use: proportion of intervention physicians who used the ARI Quality Dashboard at least once.	Not applicable	Not applicable	28%	Not applicable	Not applicable	Not applicable	Not applicable	Not applicable
Jones 2021 [10]	Peer comparison with behavioural feedback	ARI encounters in which antibiotics are	22%	23%	15.2%	23.8%	In the post-intervention period, the	-	-	Not missing data

Study ID	Intervention vs control	Outcome definition	Baseline data in Intervention group	Baseline data in control group	Measure of follow-up in Intervention group	Measure of follow-up in control group	Outcome Measure of result (changes from)			No. of missing participants
							Intervention vs control (post-intervention)	Baseline to intervention group	Baseline to control group	
	intervention vs Not intervention	inappropriate, no. (% prescribed)					difference in monthly trends in the effect of the intervention between groups was negative and significant (−0.009; 95% CI, −0.013 to −0.004; P < .001), reflecting a greater decrease in the prescribing rate in the intervention group			
		ARI encounters in which antibiotics are or may be appropriate, no. (% prescribed)	66.4%	70%	62.2%	70.1%	-	-	-	Not missing data
Davidson 2022 [13]	CHOSEN program Changes in antibiotics inappropriately prescribed between preintervention	The proportion of encounters with antibiotics inappropriately prescribed (total)	47.5% preintervention period	NA	38.7% during the intervention period	NA	The relative difference in prescribing rates was −18.5%. (P < .01). In segmented regression analysis, different level changes and slope changes were observed across each primary-care service lines. Family medicine and internal			Not missing data

Study ID	Intervention vs control	Outcome definition	Baseline data in Intervention group	Baseline data in control group	Measure of follow-up in Intervention group	Measure of follow-up in control group	Outcome Measure of result (changes from)			No. of missing participants
							Intervention vs control (post-intervention)	Baseline to intervention group	Baseline to control group	
	and intervention periods (interrupted time series (ITS) analysis)						medicine had similar patterns of change, with each showing a statistically significant level change from preintervention to intervention (family medicine, −7.95; 95% CI, −11.05 to 4.85; internal medicine, −4.73; 95% CI, −7.75 to −1.71) but similar month- to-month changes in the intervention period relative to preintervention trends.<br>Pre–Post Relative Difference in Rates, %:<br>-Family medicine: -20.4<br>-Internal medicine: -19.5<br>-Urgent care: -16.6<br>- Pediatrics: -17.2			
		β-lactamase inhibitors	17.6%	NA	19.4	NA	NR			
		Cephalosporins	14.1%	NA	14.6	NA	NR			
		Macrolides	26.9%	NA	22.1	NA	NR			
		Penicillins	27.8%	NA	27.9	NA	NR			
		Fluoroquinolones	4.5%	NA	3	NA	NR			
		Tetracyclines	7.7%	NA	11.6	NA	NR			

* There were calculated in review manager 5.4.1 with n° and % reported in the study.
NA: not applicable
NR: not report

References:

1. Du Yan, L.; Dean, K.; Park, D.; Thompson, J.; Tong, I.; Liu, C.; Hamdy, R.F. Education vs Clinician Feedback on Antibiotic Prescriptions for Acute Respiratory Infections in Telemedicine: A Randomized Controlled Trial. *J. Gen. Intern. Med.* **2021**, *36*, 305–312, doi:10.1007/s11606-020-06134-0.

2. Hemkens, L.G.; Saccilotto, R.; Reyes, S.L.; Glinz, D.; Zumbrunn, T.; Grolimund, O.; Gloy, V.; Raatz, H.; Widmer, A.; Zeller, A.; et al. Personalized Prescription Feedback to Reduce Antibiotic Overuse in Primary Care : Rationale and Design of a Nationwide Pragmatic Randomized Trial. *BMC Infect. Dis.* **2016**, 1–8, doi:10.1186/s12879-016-1739-0.

3. Hemkens, L.G.; Saccilotto, R.; Reyes, S.L.; Glinz, D.; Zumbrunn, T.; Grolimund, O.; Gloy, V.; Raatz, H.; Widmer, A.; Zeller, A.; et al. Personalized Prescription Feedback Using Routinely Collected Data to Reduce Antibiotic Use in Primary Care: A Randomized Clinical Trial. *JAMA Intern. Med.* **2017**, *177*, 176–183, doi:10.1001/jamainternmed.2016.8040.

4. Curtis, H.J.; Bacon, S.; Croker, R.; Walker, A.J.; Perera, R.; Hallsworth, M.; Harper, H.; Mahtani, K.R.; Heneghan, C.; Goldacre, B. Evaluating the Impact of a Very Low-Cost Intervention to Increase Practices' Engagement with Data and Change Prescribing Behaviour: A Randomized Trial in English Primary Care. *Fam. Pract.* **2021**, *38*, 373–380, doi:10.1093/fampra/cmaa128.

5. Linder, J.A.; Schnipper, J.L.; Tsurikova, R.; Yu, D.T.; Volk, L.A.; Melnikas, A.J.; Palchuk, M.B.; Olsha-Yehiav, M.; Middleton, B. Electronic Health Record Feedback to Improve Antibiotic Prescribing for Acute Respiratory Infections. *Am. J. Manag. Care* **2010**, *16*, 311–319.

6. Shen, X.R.; Lu, M.; Feng, R.; Cheng, J.; Chai, J.; Xie, M.; Dong, X.; Jiang, T.; Wang, D. Web-Based Just-in-Time Information and Feedback on Antibiotic Use for Village Doctors in Rural Anhui, China: Randomized Controlled Trial. *J. Med. Internet Res.* **2018**, *20*, doi:10.2196/jmir.8922.

7. Prior, M.; Elouafkaoui, P.; Elders, A.; Young, L.; Duncan, E.M.; Newlands, R.; Clarkson, J.E.; Ramsay, C.R.; Black, I.; Bonetti, D.; et al. Evaluating an Audit and Feedback Intervention for Reducing Antibiotic Prescribing Behaviour in General Dental Practice (the RAPiD Trial): A Partial Factorial Cluster Randomised Trial Protocol. *Implement. Sci.* **2014**, *9*, 1–10, doi:10.1186/1748-5908-9-50.

8. Elouafkaoui, P.; Young, L.; Newlands, R.; Duncan, E.M.; Elders, A.; Clarkson, J.E.; Ramsay, C.R. An Audit and Feedback Intervention for Reducing Antibiotic Prescribing in General Dental Practice: The RAPiD Cluster Randomised Controlled Trial. *PLoS Med.* **2016**, *13*, 1–19, doi:10.1371/journal.pmed.1002115.

9. Chang, Y.; Sangthong, R.; McNeil, E.B.; Tang, L.; Chongsuvivatwong, V. Effect of a Computer Network-Based Feedback Program on Antibiotic Prescription Rates of Primary Care Physicians: A Cluster Randomized Crossover-Controlled Trial. *J. Infect. Public Health* **2020**, *13*, 1297–1303, doi:10.1016/j.jiph.2020.05.027.

10. Jones, G.F.; Fabre, V.; Hinson, J.; Levin, S.; Toerper, M.; Townsend, J.; Cosgrove, S.E.; Saheed, M.; Klein, E.Y. Improving Antimicrobial Prescribing for Upper Respiratory Infections in the Emergency Department: Implementation of Peer Comparison with Behavioral Feedback. *Antimicrob. Steward. Healthc. Epidemiol.* **2021**, *1*, 1–6, doi:10.1017/ash.2021.240.

11. Daneman, N.; Lee, S.M.; Bai, H.; Bell, C.M.; Bronskill, S.E.; Campitelli, M.A.; Dobell, G.; Fu, L.; Garber, G.; Ivers, N.; et al. Population-Wide Peer

Comparison Audit and Feedback to Reduce Antibiotic Initiation and Duration in Long-Term Care Facilities with Embedded Randomized Controlled Trial. *Clin. Infect. Dis.* **2021**, *73*, e1296–e1304, doi:10.1093/cid/ciab256.

12. Laur, C.; Sribaskaran, T.; Simeoni, M.; Desveaux, L.; Daneman, N.; Mulhall, C.; Lam, J.; Ivers, N.M. Improving Antibiotic Initiation and Duration Prescribing among Nursing Home Physicians Using an Audit and Feedback Intervention: A Theory-Informed Qualitative Analysis. *BMJ Open Qual.* **2021**, *10*, 1–9, doi:10.1136/bmjoq-2020-001088.

13. Davidson, L.E.; Gentry, E.M.; Priem, J.S.; Kowalkowski, M.; Spencer, M.D. A Multimodal Intervention to Decrease Inappropriate Outpatient Antibiotic Prescribing for Upper Respiratory Tract Infections in a Large Integrated Healthcare System. *Infect. Control Hosp. Epidemiol.* **2022**, 1–8, doi:10.1017/ice.2022.83.
