# Supplementary material for: The Effectiveness of Interactive Dashboards to Optimise Antibiotic Prescribing in Primary Care: A Systematic Review
Source: Antibiotics (Basel). 2023 Jan 10;12(1):136. doi: 10.3390/antibiotics12010136 (PMC9854857; doi:10.3390/antibiotics12010136)
Supplement: Supplementary file 1 [file antibiotics-12-00136-s001.zip › Table S4 - Risk of bias in included studies.pdf]

Table S4. Risk of bias in included studies

**Title:** An Audit and Feedback Intervention for Reducing Antibiotic Prescribing in General Dental Practice: The RAPiD Cluster Randomised Controlled Trial		
**Study ID:** Elouafkaoui 2016 (1,2)	**Final decision**	**Comments**
**Domain 1a: Risk of bias arising from the randomization process**		
1a.1 Was the allocation sequence random?	Yes	Quote="allocation schedule for random assignment was computer generated"
1a.2 Was the allocation sequence concealed until clusters were enrolled and assigned to interventions?	Probably yes	Quote="The statistician was blinded to the identity of the practices"
1a.3 Did baseline differences between intervention groups suggest a problem with the randomization process?	Probably No	Quote = "They stratified by single-handed/ multi-handed practices and I don't observe big differents table 1, except broad spectrum antibiotics"
**Domain 1b: Risk of bias arising from the timing of identification or recruitment of participants in a cluster-randomized trial**		
1b.1 Were all the individual participants identified and recruited (if appropriate) before randomization of clusters?	Probably yes	It mentions recruitment of interviews but not for trial.
1b.2 **If N/PN/NI to 1b.1: Is it likely that selection of individual participants was affected by knowledge of the intervention assigned to the cluster?**	Not applicable	
1b.3 Were there baseline imbalances that suggest differential identification or recruitment of individual participants between intervention groups?	Probably No	We did not identify baseline imbalances (table 1)
**Domain 2: Risk of bias due to deviations from the intended interventions (effect of assignment to intervention)**		
2.1a Were participants aware that they were in a trial?	Probably yes	
2.1b. **If Y/PY/NI to 2.1a: Were participants aware of their assigned intervention during the trial?**	Probably yes	Tool said: "Answer 'Yes' if participants were aware of any part of the assigned intervention during the trial." We answered probably yes last question and I know the article is not explicit but probably participants were aware this because the article only mentioned blinding to statitician.
2.2. Were carers and people delivering the interventions aware of participants' assigned intervention during the trial?	Probably yes	Article is not explicit but probably carers and people delivering the interventions were aware this because the

		article only mentioned blinding to statitician.
2.3. **If Y/PY/NI to 2.1b or 2.2: Were there deviations from the intended intervention that arose because of the trial context?**	No Information	Deviations from the intended intervention that arise due to the trial context are rarely reported in cluster-randomized trials and may, in fact, occur rarely.
2.4 **If Y/PY to 2.3: Were these deviations likely to have affected the outcome?**	Not applicable	
2.5. **If Y/PY/NI to 2.4: Were these deviations from intended intervention balanced between groups?**	Not applicable	
2.6 Was an appropriate analysis used to estimate the effect of assignment to intervention?	Probably yes	We considered "probably yes", because the analyses were clustered by dental practice, and they wer adjusted for clustering of dentists<br>within practice using the Huber-White robust standard error procedure
2.7 **If N/PN/NI to 2.6: Was there potential for a substantial impact (on the result) of the failure to analyse participants in the group to which they were randomized?**	Not applicable	
**Domain 3: Risk of bias due to missing outcome data**		
3.1a Were data for this outcome available for all clusters that recruited participants?	Yes	
3.1b Were data for this outcome available for all, or nearly all, participants within clusters?	Probably yes	
3.2 **If N/PN/NI to 3.1a or 3.1b: Is there evidence that the result was not biased by missing data?**	Not applicable	
3.3 **If N/PN to 3.2 Could missingness in the outcome depend on its true value?**	Not applicable	
3.4 **If Y/PY/NI to 3.3: Is it likely that missingness in the outcome depended on its true value?**	Not applicable	
**Domain 4: Risk of bias in measurement of the outcome**		
4.1 Was the method of measuring the outcome inappropriate?	No	These are pre-specified outcomes (not sensible) and were measured appropriate
4.2 Could measurement or ascertainment of the outcome have differed between intervention groups?	Probably No	We did not identify any difference between groups.

4.3a If N/PN/NI to 4.1 and 4.2: **Were outcome assessors aware that a trial was taking place?**	Probably No	Quote="The statistician was blinded to the identity of the practices"
4.3b **If Y/PY/NI to 4.3a: Were outcome assessors aware of the intervention received by study participants?**	Not applicable	
4.4 **If Y/PY/NI to 4.3b: Could assessment of the outcome have been influenced by knowledge of intervention received?**	Not applicable	
4.5 If **Y/PY/NI to 4.4: Is it likely that assessment of the outcome was influenced by knowledge of intervention received?**	Not applicable	
**Domain 5: Risk of bias in selection of the reported result**		
5.1 Were the data that produced this result analysed in accordance with a pre-specified analysis plan that was finalized before unblinded outcome data were available for analysis?	Probably yes	
Is the numerical result being assessed likely to have been selected, on the basis of the results, from...		
5.2. ... multiple eligible outcome measurements (e.g. scales, definitions, time points) within the outcome domain?	Probably No	They analyzed each at multiple time points.<br>There is clear evidence that all eligible reported results for the outcome domain correspond to all intended outcome measurements.
5.3 ... multiple eligible analyses of the data?	Probably No	
Tool: Sandra Eldridge, Marion K Campbell, Michael J Campbell, Amy K Drahota, Bruno Giraudeau, Barnaby C Reeves, Nandi Siegfried, Julian PT Higgins. Additional considerations for cluster-randomized trials (RoB 2 CRT). March 2021. Available in: https://sites.google.com/site/riskofbiastool/welcome/rob-2-0-tool/rob-2-for-cluster-randomized-trials		

**Title:** Evaluating the impact of a very low-cost intervention to increase practices' engagement with data and change prescribing behaviour: a randomized trial in English primary care		
**Study ID:** Curtis 2021 (3)	**Final decision**	**Comments**
**Domain 1a: Risk of bias arising from the randomization process**		
1a.1 Was the allocation sequence random?	Yes	
1a.2 Was the allocation sequence concealed until clusters were enrolled and assigned to interventions?	Probably yes	
1a.3 Did baseline differences between intervention groups suggest a problem with the randomization process?	No	We did not identify baseline imbalances (table 1)

**Domain 1b: Risk of bias arising from the timing of identification or recruitment of participants in a cluster-randomized trial**		
1b.1 Were all the individual participants identified and recruited (if appropriate) before randomization of clusters?	Yes	See in participants section
1b.2 **If N/PN/NI to 1b.1: Is it likely that selection of individual participants was affected by knowledge of the intervention assigned to the cluster?**	Not applicable	
1b.3 Were there baseline imbalances that suggest differential identification or recruitment of individual participants between intervention groups?	Probably No	We did not identify baseline imbalances (table 1) and protocol
**Domain 2: Risk of bias due to deviations from the intended interventions (effect of assignment to intervention)**		
2.1a Were participants aware that they were in a trial?	Probably No	Quote="Participants were not informed that they were in a trial"
2.1b. **If Y/PY/NI to 2.1a: Were participants aware of their assigned intervention during the trial?**	Not applicable	
2.2. Were carers and people delivering the interventions aware of participants' assigned intervention during the trial?	Probably yes	Article is not explicit but probably carers and people delivering the interventions were aware this because the article did not mention blinding process
2.3. **If Y/PY/NI to 2.1b or 2.2: Were there deviations from the intended intervention that arose because of the trial context?**	No Information	Deviations from the intended intervention that arise due to the trial context are rarely reported in cluster-randomized trials and may, in fact, occur rarely.
2.4 **If Y/PY to 2.3: Were these deviations likely to have affected the outcome?**	Not applicable	
2.5. **If Y/PY/NI to 2.4: Were these deviations from intended intervention balanced between groups?**	Not applicable	
2.6 Was an appropriate analysis used to estimate the effect of assignment to intervention?	Probably yes	They justified the statistical analysis in their protocol
2.7 **If N/PN/NI to 2.6: Was there potential for a substantial impact (on the result) of the failure to analyse participants in the group to which they were randomized?**	Not applicable	
**Domain 3: Risk of bias due to missing outcome data**		

3.1a Were data for this outcome available for all clusters that recruited participants?	Probably yes	Cluster analysis and they reported information of all cluster.
3.1b Were data for this outcome available for all, or nearly all, participants within clusters?	Probably yes	Cluster analysis and they reported information of all cluster.
3.2 **If N/PN/NI to 3.1a or 3.1b: Is there evidence that the result was not biased by missing data?**	Not applicable	
3.3 **If N/PN to 3.2 Could missingness in the outcome depend on its true value?**	Not applicable	
3.4 **If Y/PY/NI to 3.3: Is it likely that missingness in the outcome depended on its true value?**	Not applicable	
**Domain 4: Risk of bias in measurement of the outcome**		
4.1 Was the method of measuring the outcome inappropriate?	Probably No	These are pre-specified outcomes (not sensible) and were measured appropriate
4.2 Could measurement or ascertainment of the outcome have differed between intervention groups?	Probably No	Our interest outcome is antibiotic prescribing, and this did not differ between groups.
4.3a If **N/PN/NI to 4.1 and 4.2: Were outcome assessors aware that a trial was taking place?**	No Information	
4.3b **If Y/PY/NI to 4.3a: Were outcome assessors aware of the intervention received by study participants?**	Probably yes	They did not mention blinding process on outcome assessors
4.4 **If Y/PY/NI to 4.3b: Could assessment of the outcome have been influenced by knowledge of intervention received?**	No	
4.5 If **Y/PY/NI to 4.4: Is it likely that assessment of the outcome was influenced by knowledge of intervention received?**	Not applicable	
**Domain 5: Risk of bias in selection of the reported result**		
5.1 Were the data that produced this result analysed in accordance with a pre-specified analysis plan that was finalized before unblinded outcome data were available for analysis?	Yes	
Is the numerical result being assessed likely to have been selected, on the basis of the results, from...		
5.2. ... multiple eligible outcome measurements (e.g. scales, definitions, time points) within the outcome domain?	Probably No	There is clear evidence that all eligible reported results for the outcome domain correspond to all intended outcome measurements.
5.3 ... multiple eligible analyses of the data?	Probably No	

Tool: Sandra Eldridge, Marion K Campbell, Michael J Campbell, Amy K Drahota, Bruno Giraudeau, Barnaby C Reeves, Nandi Siegfried, Julian PT Higgins. Additional considerations for cluster-randomized trials (RoB 2 CRT). March 2021. Available in: https://sites.google.com/site/riskofbiastool/welcome/rob-2-0-tool/rob-2-for-cluster-randomized-trials

---

**Title:** Electronic health record feedback to improve antibiotic prescribing for acute respiratory infections		
**Study ID:** Linder 2010 (4)	**Final decision**	**Comments**
**Domain 1a: Risk of bias arising from the randomization process**		
1a.1 Was the allocation sequence random?	Probably yes	We observed only information about randomization methods is a statement that the study is randomized.
1a.2 Was the allocation sequence concealed until clusters were enrolled and assigned to interventions?	No Information	
1a.3 Did baseline differences between intervention groups suggest a problem with the randomization process?	Probably No	Quote="There was no significant difference between intervention and control practices in number of years using the EHR, mean visits per year, the baseline antibiotic prescribing rate, or the baseline antibiotic prescribing rate for ARIs (data not shown)"
**Domain 1b: Risk of bias arising from the timing of identification or recruitment of participants in a cluster-randomized trial**		
1b.1 Were all the individual participants identified and recruited (if appropriate) before randomization of clusters?	Probably yes	
1b.2 **If N/PN/NI to 1b.1: Is it likely that selection of individual participants was affected by knowledge of the intervention assigned to the cluster?**	Not applicable	
1b.3 Were there baseline imbalances that suggest differential identification or recruitment of individual participants between intervention groups?	No	
**Domain 2: Risk of bias due to deviations from the intended interventions (effect of assignment to intervention)**		
2.1a Were participants aware that they were in a trial?	Probably yes	They only mentioned "Study investigators were blinded to the randomization." but not participants
2.1b. **If Y/PY/NI to 2.1a: Were participants aware of their assigned intervention during the trial?**	No	

2.2. Were carers and people delivering the interventions aware of participants' assigned intervention during the trial?	Probably No	Quote="Study investigators were blinded to the randomization."
2.3. **If Y/PY/NI to 2.1b or 2.2: Were there deviations from the intended intervention that arose because of the trial context?**	Not applicable	
2.4 **If Y/PY to 2.3: Were these deviations likely to have affected the outcome?**	Not applicable	
2.5. **If Y/PY/NI to 2.4: Were these deviations from intended intervention balanced between groups?**	Not applicable	
2.6 Was an appropriate analysis used to estimate the effect of assignment to intervention?	Yes	
2.7 **If N/PN/NI to 2.6: Was there potential for a substantial impact (on the result) of the failure to analyse participants in the group to which they were randomized?**	Not applicable	
**Domain 3: Risk of bias due to missing outcome data**		
3.1a Were data for this outcome available for all clusters that recruited participants?	Probably yes	Cluster analysis and they reported information of all cluster.
3.1b Were data for this outcome available for all, or nearly all, participants within clusters?	Probably yes	Cluster analysis and they reported information of all cluster.
3.2 **If N/PN/NI to 3.1a or 3.1b: Is there evidence that the result was not biased by missing data?**	Not applicable	
3.3 **If N/PN to 3.2 Could missingness in the outcome depend on its true value?**	Not applicable	
3.4 **If Y/PY/NI to 3.3: Is it likely that missingness in the outcome depended on its true value?**	Not applicable	
**Domain 4: Risk of bias in measurement of the outcome**		
4.1 Was the method of measuring the outcome inappropriate?	Probably No	
4.2 Could measurement or ascertainment of the outcome have differed between intervention groups?	Probably No	We did not identify any difference between groups.
4.3a If **N/PN/NI to 4.1 and 4.2: Were outcome assessors aware that a trial was taking place?**	Probably yes	They only mentioned "Study investigators were blinded to the randomization." but not outcome assessors
4.3b **If Y/PY/NI to 4.3a: Were outcome assessors aware of the intervention received by study participants?**	No Information	

4.4 **If Y/PY/NI to 4.3b: Could assessment of the outcome have been influenced by knowledge of intervention received?**	Probably No	
4.5 If **Y/PY/NI to 4.4: Is it likely that assessment of the outcome was influenced by knowledge of intervention received?**	Not applicable	
**Domain 5: Risk of bias in selection of the reported result**		
5.1 Were the data that produced this result analysed in accordance with a pre-specified analysis plan that was finalized before unblinded outcome data were available for analysis?	No Information	We do not have access to protocol to evaluate pre-specified analysis
Is the numerical result being assessed likely to have been selected, on the basis of the results, from...		
5.2. ... multiple eligible outcome measurements (e.g. scales, definitions, time points) within the outcome domain?	Probably No	There is clear evidence that all eligible reported results for the outcome domain correspond to all intended outcome measurements.
5.3 ... multiple eligible analyses of the data?	Probably No	
Tool: Sandra Eldridge, Marion K Campbell, Michael J Campbell, Amy K Drahota, Bruno Giraudeau, Barnaby C Reeves, Nandi Siegfried, Julian PT Higgins. Additional considerations for cluster-randomized trials (RoB 2 CRT). March 2021. Available in: https://sites.google.com/site/riskofbiastool/welcome/rob-2-0-tool/rob-2-for-cluster-randomized-trials		

**Title:** Web-based just-in-time information and feedback on antibiotic use for village doctors in rural Anhui, China: Randomized controlled trial		
**Study ID:** Shen XR 2018 (5)	**Final decision**	**Comments**
**Domain 1a: Risk of bias arising from the randomization process**		
1a.1 Was the allocation sequence random?	No information	Comment= the only information about randomization methods was a statement that the study is randomized. It never mentioned the sequence generation process.
1a.2 Was the allocation sequence concealed until clusters were enrolled and assigned to interventions?	Probably no	Comment= Probably the enrolling investigator or the participant had knowledge of the forthcoming<br>Allocation because it is unclear the allocation sequence concealed
1a.3 Did baseline differences between intervention groups suggest a problem with the randomization process?	No	Comment= Table 1, it did not observe imbalances
**Domain 1b: Risk of bias arising from the timing of identification or recruitment of participants in a cluster-randomized trial**		

1b.1 Were all the individual participants identified and recruited (if appropriate) before randomization of clusters?	Yes	Comment=individual participants were not recruited at all, but all were identified before randomization
1b.2 **If N/PN/NI to 1b.1: Is it likely that selection of individual participants was affected by knowledge of the intervention assigned to the cluster?**	Not applicable	
1b.3 Were there baseline imbalances that suggest differential identification or recruitment of individual participants between intervention groups?	Probably no	Quote= "*The study sites were determined via a 3-step clustered randomization*"
**Domain 2: Risk of bias due to deviations from the intended interventions (effect of assignment to intervention)**		
2.1a Were participants aware that they were in a trial?	Probably yes	Quote=" *the biggest concern of the study may be observation-induced interferences on the practice behaviors.*" Comments=it is not blinding
2.1b. **If Y/PY/NI to 2.1a: Were participants aware of their assigned intervention during the trial?**	Probably yes	Quote=" *doctors on the intervention arm were given detailed references, SOPs, and feedback, and thus they knew much better about what they were expected to do than those in the control group.*"
2.2. Were carers and people delivering the interventions aware of participants' assigned intervention during the trial?	Probably yes	Quote=" *the biggest concern of the study may be observation-induced interferences on the practice behaviors.*" Comments=it is not blinding
2.3. **If Y/PY/NI to 2.1b or 2.2: Were there deviations from the intended intervention that arose because of the trial context?**	Probably no	
2.4 **If Y/PY to 2.3: Were these deviations likely to have affected the outcome?**	Not applicable	
2.5. **If Y/PY/NI to 2.4: Were these deviations from intended intervention balanced between groups?**	Not applicable	
2.6 Was an appropriate analysis used to estimate the effect of assignment to intervention?	Yes	
2.7 **If N/PN/NI to 2.6: Was there potential for a substantial impact (on the result) of the failure to analyse participants in the group to which they were randomized?**	Not applicable	
**Domain 3: Risk of bias due to missing outcome data**		

3.1a Were data for this outcome available for all clusters that recruited participants?	Probably yes	
3.1b Were data for this outcome available for all, or nearly all, participants within clusters?	Probably yes	Missing data=5% of patients in intervention group – baseline<br>4% of patients in intervention group – baseline<br>3% of patients in intervention group – endpoint<br>4% of patients in intervention group – endpoint
3.2 **If N/PN/NI to 3.1a or 3.1b: Is there evidence that the result was not biased by missing data?**	Not applicable	
3.3 **If N/PN to 3.2 Could missingness in the outcome depend on its true value?**	Not applicable	
3.4 **If Y/PY/NI to 3.3: Is it likely that missingness in the outcome depended on its true value?**	Not applicable	
**Domain 4: Risk of bias in measurement of the outcome**		
4.1 Was the method of measuring the outcome inappropriate?	Probably no	
4.2 Could measurement or ascertainment of the outcome have differed between intervention groups?	Probably no	
4.3a If **N/PN/NI to 4.1 and 4.2: Were outcome assessors aware that a trial was taking place?**	Probably yes	Quote=" *the biggest concern of the study may be observation-induced interferences on the practice behaviors*."<br>Comments=it is not blinding
4.3b **If Y/PY/NI to 4.3a: Were outcome assessors aware of the intervention received by study participants?**	Probably yes	
4.4 **If Y/PY/NI to 4.3b: Could assessment of the outcome have been influenced by knowledge of intervention received?**	Probably yes	Quote="*The field data collectors may have given, due to various reasons, more positive ratings to intervention than the control groups since they knew the grouping, though the combination of the data quality control measures may have helped in keeping to a minimum*."
4.5 If **Y/PY/NI to 4.4: Is it likely that assessment of the outcome was influenced by knowledge of intervention received?**	No Information	Comments= there is no reason to believe that it did
**Domain 5: Risk of bias in selection of the reported result**		

5.1 Were the data that produced this result analysed in accordance with a pre-specified analysis plan that was finalized before unblinded outcome data were available for analysis?	No Information	We do not have access to protocol to evaluate pre-specified analysis
Is the numerical result being assessed likely to have been selected, on the basis of the results, from...		
5.2. ... multiple eligible outcome measurements (e.g. scales, definitions, time points) within the outcome domain?	Probably No	There is clear evidence that all eligible reported results for the outcome domain correspond to all intended outcome measurements.
5.3 ... multiple eligible analyses of the data?	Probably No	
Tool: Sandra Eldridge, Marion K Campbell, Michael J Campbell, Amy K Drahota, Bruno Giraudeau, Barnaby C Reeves, Nandi Siegfried, Julian PT Higgins. Additional considerations for cluster-randomized trials (RoB 2 CRT). March 2021. Available in: https://sites.google.com/site/riskofbiastool/welcome/rob-2-0-tool/rob-2-for-cluster-randomized-trials		

**Title:** Education vs Clinician Feedback on Antibiotic Prescriptions for Acute Respiratory Infections in Telemedicine: a Randomized Controlled Trial		
**Study ID:** Du Yan 2021 (6)	**Final decision**	**Comments**
**Domain 1a: Risk of bias arising from the randomization process**		
1.1 Was the allocation sequence random?	Probably yes	Quote="A programming engineer not involved in the study randomized clinicians using a randomization sorting function."
1.2 Was the allocation sequence concealed until participants were enrolled and assigned to interventions?	Yes	Quote= "Study investigators were blinded to the randomization."
1.3 Did baseline differences between intervention groups suggest a problem with the randomization process?	No	Not imbalances are apparent
**Domain 2: Risk of bias due to deviations from the intended interventions (effect of assignment to intervention)**		
2.1. Were participants aware of their assigned intervention during the trial?	Probably yes	Participants were not blinded
2.2. Were carers and people delivering the interventions aware of participants' assigned intervention during the trial?	Probably No	Quote= "Study investigators were blinded to the randomization."
2.3. **If Y/PY/NI to 2.1 or 2.2: Were there deviations from the intended intervention that arose because of the trial context?**	No Information	We did not have access to trial protocol
2.4 **If Y/PY to 2.3: Were these deviations likely to have affected the outcome?**	Not applicable	

2.5. **If Y/PY/NI to 2.4: Were these deviations from intended intervention balanced between groups?**	Not applicable	
2.6 Was an appropriate analysis used to estimate the effect of assignment to intervention?	Probably yes	Quote="We used intention-to-treat analysis."
2.7 **If N/PN/NI to 2.6: Was there potential for a substantial impact (on the result) of the failure to analyse participants in the group to which they were randomized?**	Not applicable	
**Domain 3: Risk of bias due to missing outcome data**		
3.1 Were data for this outcome available for all, or nearly all, participants randomized?	Yes	
3.2 **If N/PN/NI to 3.1: Is there evidence that the result was not biased by missing outcome data?**	Not applicable	
3.3 **If N/PN to 3.2: Could missingness in the outcome depend on its true value?**	Not applicable	
3.4 **If Y/PY/NI to 3.3: Is it likely that missingness in the outcome depended on its true value?**	Not applicable	
**Domain 4: Risk of bias in measurement of the outcome**		
4.1 Was the method of measuring the outcome inappropriate?	Probably No	
4.2 Could measurement or ascertainment of the outcome have differed between intervention groups?	Probably No	
4.3 **If N/PN/NI to 4.1 and 4.2: Were outcome assessors aware of the intervention received by study participants?**	Probably yes	Article is not explicit but they only mentioned "Study investigators were blinded to the randomization", probably outcome assessors know about intervention
4.4 **If Y/PY/NI to 4.3: Could assessment of the outcome have been influenced by knowledge of intervention received?**	Probably yes	
4.5 **If Y/PY/NI to 4.4: Is it likely that assessment of the outcome was influenced by knowledge of intervention received?**	Probably No	
**Domain 5: Risk of bias in selection of the reported result**		
5.1 Were the data that produced this result analysed in accordance with a pre-specified analysis plan that was finalized before unblinded outcome data were available for analysis?	No Information	We did not have access to trial protocol
Is the numerical result being assessed likely to have been selected, on the basis of the results, from...		

5.2. ... multiple eligible outcome measurements (e.g. scales, definitions, time points) within the outcome domain?	Probably No	There is clear evidence that all eligible reported results for the outcome domain correspond to all intended outcome measurements.
5.3 ... multiple eligible analyses of the data?	Probably No	
Tool: Julian PT Higgins, Jelena Savović, Matthew J Page, Jonathan AC Sterne. Revised Cochrane risk-of-bias tool for randomized trials (RoB 2). August 2019. Available in: https://sites.google.com/site/riskofbiastool/welcome/rob-2-0-tool/current-version-of-rob-2		

**Title:** Population-Wide Peer Comparison Audit and Feedback to Reduce Antibiotic Initiation and Duration in Long-Term Care Facilities with Embedded Randomized Controlled Trial		
**Study ID:** Daneman 2021 (7,8)	**Final decision**	**Comments**
**Domain 1a: Risk of bias arising from the randomization process**		
1.1 Was the allocation sequence random?	Yes	Randomization was performed without stratification via random sequence generation centrally at ICES using encoded physician registration numbers
1.2 Was the allocation sequence concealed until participants were enrolled and assigned to interventions?	Yes	Allocation was concealed centrally and then revealed only to the Ontario Health team that disseminated the reports.
1.3 Did baseline differences between intervention groups suggest a problem with the randomization process?	Probably No	Not imbalances are apparent
**Domain 2: Risk of bias due to deviations from the intended interventions (effect of assignment to intervention)**		
2.1. Were participants aware of their assigned intervention during the trial?	Probably yes	Quote="Participating physicians were, by definition, aware of the type of report they had received, but the residents they cared for were unaware."
2.2. Were carers and people delivering the interventions aware of participants' assigned intervention during the trial?	No	
2.3. **If Y/PY/NI to 2.1 or 2.2: Were there deviations from the intended intervention that arose because of the trial context?**	No	
2.4 **If Y/PY to 2.3: Were these deviations likely to have affected the outcome?**	Not applicable	
2.5. **If Y/PY/NI to 2.4: Were these deviations from intended intervention balanced between groups?**	Not applicable	

2.6 Was an appropriate analysis used to estimate the effect of assignment to intervention?	Yes	The embedded trial of report format was evaluated using an intention to treat approach.
2.7 **If N/PN/NI to 2.6: Was there potential for a substantial impact (on the result) of the failure to analyse participants in the group to which they were randomized?**	Not applicable	
**Domain 3: Risk of bias due to missing outcome data**		
3.1 Were data for this outcome available for all, or nearly all, participants randomized?	Yes	The embedded trial of report format was evaluated using an intention to treat approach.
3.2 **If N/PN/NI to 3.1: Is there evidence that the result was not biased by missing outcome data?**	Not applicable	
3.3 **If N/PN to 3.2: Could missingness in the outcome depend on its true value?**	Not applicable	
3.4 **If Y/PY/NI to 3.3: Is it likely that missingness in the outcome depended on its true value?**	Not applicable	
**Domain 4: Risk of bias in measurement of the outcome**		
4.1 Was the method of measuring the outcome inappropriate?	Probably No	
4.2 Could measurement or ascertainment of the outcome have differed between intervention groups?	Probably No	We did not identify any difference between groups.
4.3 **If N/PN/NI to 4.1 and 4.2: Were outcome assessors aware of the intervention received by study participants?**	Probably yes	The study team at ICES remained blinded to treatment allocation until completion of analyses.<br>Clinicaltrials https://clinicaltrials.gov/ct2/show/NCT03807466: masking Triple (Participant, Investigator, Outcomes Assessor)
4.4 **If Y/PY/NI to 4.3: Could assessment of the outcome have been influenced by knowledge of intervention received?**	Probably No	
4.5 **If Y/PY/NI to 4.4: Is it likely that assessment of the outcome was influenced by knowledge of intervention received?**	Not applicable	
**Domain 5: Risk of bias in selection of the reported result**		
5.1 Were the data that produced this result analysed in accordance with a pre-specified analysis plan that was finalized before unblinded outcome data were available for analysis?	Probably yes	https://clinicaltrials.gov/ct2/show/NCT03807466
Is the numerical result being assessed likely to have been selected, on the basis of the results, from...		

5.2. ... multiple eligible outcome measurements (e.g. scales, definitions, time points) within the outcome domain?	Probably No	There is clear evidence that all eligible reported results for the outcome domain correspond to all intended outcome measurements.
5.3 ... multiple eligible analyses of the data?	Probably No	
Tool: Julian PT Higgins, Jelena Savović, Matthew J Page, Jonathan AC Sterne. Revised Cochrane risk-of-bias tool for randomized trials (RoB 2). August 2019. Available in: https://sites.google.com/site/riskofbiastool/welcome/rob-2-0-tool/current-version-of-rob-2		

**Title:** Personalized Prescription Feedback Using Routinely Collected Data to Reduce Antibiotic Use in Primary Care: A Randomized Clinical Trial		
**Study ID:** Hemkens LG (9,10)	**Final decision**	**Comments**
**Domain 1a: Risk of bias arising from the randomization process**		
1.1 Was the allocation sequence random?	Yes	Quote=”…*using a computer algorithm*.”
1.2 Was the allocation sequence concealed until participants were enrolled and assigned to interventions?	Probably yes	Comment= independent of the enrolment personnel
1.3 Did baseline differences between intervention groups suggest a problem with the randomization process?	No	Comment= no imbalances are apparent
**Domain 2: Risk of bias due to deviations from the intended interventions (effect of assignment to intervention)**		
2.1. Were participants aware of their assigned intervention during the trial?	No	Quote=”*Physicians in the intervention group were not aware of being part of a controlled trial; physicians in the control group were not informed*”
2.2. Were carers and people delivering the interventions aware of participants' assigned intervention during the trial?	No	Quote=”*Investigators were blinded owing to the anonymized nature of the trial*.”
2.3. **If Y/PY/NI to 2.1 or 2.2: Were there deviations from the intended intervention that arose because of the trial context?**	Not applicable	
2.4 **If Y/PY to 2.3: Were these deviations likely to have affected the outcome?**	Not applicable	
2.5. **If Y/PY/NI to 2.4: Were these deviations from intended intervention balanced between groups?**	Not applicable	
2.6 Was an appropriate analysis used to estimate the effect of assignment to intervention?	Yes	Comment=they used intention-to-treat (ITT), and also did sensibility analysis
2.7 **If N/PN/NI to 2.6: Was there potential for a substantial impact (on the result) of the failure to**	Not applicable	

**analyse participants in the group to which they were randomized?**		
**Domain 3: Risk of bias due to missing outcome data**		
3.1 Were data for this outcome available for all, or nearly all, participants randomized?	Yes	Comment=analysis of the intention to treat effect is all randomized participants.
3.2 **If N/PN/NI to 3.1: Is there evidence that the result was not biased by missing outcome data?**	Not applicable	
3.3 **If N/PN to 3.2: Could missingness in the outcome depend on its true value?**	Not applicable	
3.4 **If Y/PY/NI to 3.3: Is it likely that missingness in the outcome depended on its true value?**	Not applicable	
**Domain 4: Risk of bias in measurement of the outcome**		
4.1 Was the method of measuring the outcome inappropriate?	No	
4.2 Could measurement or ascertainment of the outcome have differed between intervention groups?	No	
4.3 **If N/PN/NI to 4.1 and 4.2: Were outcome assessors aware of the intervention received by study participants?**	No	Quote= "*The outcome assessment was, formally, blinded because all study-relevant data were collected by health insurance personnel not involved in the study*"
4.4 **If Y/PY/NI to 4.3: Could assessment of the outcome have been influenced by knowledge of intervention received?**	Not applicable	
4.5 **If Y/PY/NI to 4.4: Is it likely that assessment of the outcome was influenced by knowledge of intervention received?**	Not applicable	
**Domain 5: Risk of bias in selection of the reported result**		
5.1 Were the data that produced this result analysed in accordance with a pre-specified analysis plan that was finalized before unblinded outcome data were available for analysis?	Yes	
Is the numerical result being assessed likely to have been selected, on the basis of the results, from...		
5.2. ... multiple eligible outcome measurements (e.g. scales, definitions, time points) within the outcome domain?	Probably no	There is clear evidence that all eligible reported results for the outcome domain correspond to all intended outcome measurements.
5.3 ... multiple eligible analyses of the data?	Probably no	

Tool: Julian PT Higgins, Jelena Savović, Matthew J Page, Jonathan AC Sterne. Revised Cochrane risk-of-bias tool for randomized trials (RoB 2). August 2019. Available in: https://sites.google.com/site/riskofbiastool/welcome/rob-2-0-tool/current-version-of-rob-2

**Title:** Effect of a computer network-based feedback program on antibiotic prescription rates of primary care physicians: A cluster randomized crossover-controlled trial.		
**Study ID:** Chang 2020 (11)	**Final decision**	**Comments**
**Domain 1a: Risk of bias arising from the randomization process**		
1.1 Was the allocation sequence random?	Yes	Quote= "Thirty-one township hospitals from six counties in the northern, western and southern parts of Guizhou Province were randomly selected by the information technology staff of LWTC using a computer-generated number from the list of 84 hospitals that met the criteria."
1.2 Was the allocation sequence concealed until participants were enrolled and assigned to interventions?	Probably yes	Quote= "Province were randomly selected by the information technology staff of LWTC using a computer-generated number from the list of 84 hospitals that met the criteria"
1.3 Did baseline differences between intervention groups at the start of the first period suggest a problem with the randomization process?	Probably No	The baseline characteristics of physicians were similar between the two groups in terms of age, sex, work duration, professional title, position, and education.
**Domain S: Risk of bias arising from period and carryover effects**		
S.1 Was the number of participants allocated to each of the two sequences equal or nearly equal?	Yes	pages 81 & 82 respectively
S.2 **If N/PN/NI to S.1: Were period effects accounted for in the analysis?**	Not applicable	
S.3 Was there sufficient time for any carryover effects to have disappeared before outcome assessment in the second period?	Probably No	With the comments two review, probably this study was affected by carryover effect. It did not have washout period and must have been important to implement or they selected and wrong study design to evaluate this intervention.
**Domain 2: Risk of bias due to deviations from the intended interventions (effect of assignment to intervention)**		
2.1. Were participants aware of their assigned intervention during each period of the trial?	Yes	Quote="Identification of the subjects was blinded to the investigator, but each participant automatically knew whether

		he/she was in the intervention or control group during each particular period."
2.2. Were carers and people delivering the interventions aware of participants' assigned intervention during each period of the trial?	Probably No	Quote= "Identification of the subjects was blinded to the investigator"
2.3. **If Y/PY/NI to 2.1 or 2.2: Were there deviations from the intended intervention that arose because of the trial context?**	Probably No	No report in the deviation of implementation
2.4 **If Y/PY to 2.3: Were these deviations likely to have affected the outcome?**	Not applicable	
2.5. **If Y/PY/NI to 2.4: Were these deviations from intended intervention balanced between groups?**	Not applicable	
2.6 Was an appropriate analysis used to estimate the effect of assignment to intervention?	Probably yes	
2.7 **If N/PN/NI to 2.6: Was there potential for a substantial impact (on the result) of the failure to analyse participants in the group to which they were randomized?**	Not applicable	
**Domain 3: Risk of bias due to missing outcome data**		
3.1 Were data for this outcome available for all, or nearly all, participants randomized?	Yes	Only one participant's data was not analysed, due to technical errors
3.2 **If N/PN/NI to 3.1: Is there evidence that the result was not biased by missing outcome data?**	Not applicable	
3.3 **If N/PN to 3.2: Could missingness in the outcome depend on its true value?**	Not applicable	
3.4 **If Y/PY/NI to 3.3: Is it likely that missingness in the outcome depended on its true value?**	Not applicable	
**Domain 4: Risk of bias in measurement of the outcome**		
4.1 Was the method of measuring the outcome inappropriate?	No	
4.2 Could measurement or ascertainment of the outcome have differed between interventions within each sequence?	Probably No	The same measurement methods and thresholds, used at comparable time points. Here the problem was washout period that could have affected carryover effect.
4.3 **If N/PN/NI to 4.1 and 4.2: Were outcome assessors aware of the intervention received by study participants?**	Probably No	Quote= "Identification of the subjects was blinded to the investigator"

4.4 **If Y/PY/NI to 4.3: Could assessment of the outcome have been influenced by knowledge of intervention received?**	Not applicable	
4.5 **If Y/PY/NI to 4.4: Is it likely that assessment of the outcome was influenced by knowledge of intervention received?**	Not applicable	
**Domain 5: Risk of bias in selection of the reported result**		
5.1 Were the data that produced this result analysed in accordance with a pre-specified analysis plan that was finalized before unblinded outcome data were available for analysis?	Probably yes	No report of deviation
Is the numerical result being assessed likely to have been selected, on the basis of the results, from...		
5.2. ... multiple eligible outcome measurements (e.g. scales, definitions, time points) within the outcome domain?	Probably No	
5.3 ... multiple eligible analyses of the data?	Probably No	
5.4 Is a result based on data from both periods sought, but unavailable on the basis of carryover having been identified?	No	
Tool: Julian PT Higgins, Tianjing Li and Jonathan Sterne. Additional considerations for crossover trials. March 2021. Available in: https://sites.google.com/site/riskofbiastool/welcome/rob-2-0-tool/rob-2-for-crossover-trials		

**Title:** Improving antimicrobial prescribing for upper respiratory infections in the emergency department: Implementation of peer comparison with behavioral feedback		
**Study ID:** Jones 2021 (12)	**Final decision**	**Comments**
**Domain 1: Bias due to confounding**		
1.1 Is there potential for confounding of the effect of intervention in this study?	Probably No	They included into model a seasonal covariate to account for influenza season running from November to March.
**If Y/PY to 1.1: determine whether there is a need to assess time-varying confounding:**<br>1.2. Was the analysis based on splitting participants' follow up time according to intervention received?<br>If N/PN, answer questions relating to baseline confounding (1.4 to 1.6)<br>If Y/PY, proceed to question 1.3.	Not applicable	

**If Y/PY to 1.1: determine whether there is a need to assess time-varying confounding:**<br>1.3. Were intervention discontinuations or switches likely to be related to factors that are prognostic for the outcome?<br>If N/PN, answer questions relating to baseline confounding (1.4 to 1.6)<br>If Y/PY, answer questions relating to both baseline and time-varying confounding (1.7 and 1.8)	Not applicable	
1.4. Did the authors use an appropriate analysis method that<br>controlled for all the important confounding domains?	Probably yes	Quote= "They controlled for monthly ARI encounters and included a seasonal covariate to account for influenza season running from November to March"
**1.5. If Y/PY to 1.4: Were confounding domains that were controlled for measured validly and reliably by the variables available in this study?**	Probably yes	There are not subjective measures. "They controlled for monthly ARI encounters and included a seasonal covariate to account for influenza season running from November to March"
1.6. Did the authors control for any post-intervention variables that could have been affected by the intervention?	No	We did not identify any controlling for mediating variable post-intervention.
1.7. Did the authors use an appropriate analysis method that<br>adjusted for all the important confounding domains and for time-varying confounding?	Probably yes	See table 4 "seasonal covariate"
**1.8. If Y/PY to 1.7: Were confounding domains that were adjusted for measured validly and reliably by the variables available in this study?**	Probably yes	There are not subjective measures.
Risk of bias judgement	Moderate	(i) Confounding expected, all known important confounding domains appropriately measured and controlled for
**Bias in selection of participants into the study**		
2.1. Was selection of participants into the study (or into the analysis) based on participant characteristics observed after the<br>start of intervention? **If N/PN to 2.1: go to 2.4.**	Probably No	Participants and variables measurement were selected before the start of intervention. See Study design, setting, and population

**2.2. If Y/PY to 2.1: Were the post-intervention variables that influenced selection likely to be associated with intervention?**	Not applicable	
**2.3 If Y/PY to 2.2: Were the post-intervention variables that influenced selection likely to be influenced by the outcome or a cause of the outcome?**	Not applicable	
2.4. Do start of follow-up and start of intervention coincide for most participants?	Probably yes	Similar period is mentioned in the paper
**2.5. If Y/PY to 2.2 and 2.3, or N/PN to 2.4: Were adjustment techniques used that are likely to correct for the presence of selection biases?**	Not applicable	
Risk of bias judgement	Low	
**Bias in classification of interventions**		
3.1 Were intervention groups clearly defined?	Yes	Details provided in the table 1
3.2 Was the information used to define intervention groups recorded at the start of the intervention?	Yes	Details provided in the table 1
3.3 Could classification of intervention status have been affected by knowledge of the outcome or risk of the outcome?	Probably No	No reported change in the status during the intervention
Risk of bias judgement	Low	No reported changes or modification on the intervention groups
**Bias due to deviations from intended interventions**		
4.1. Were there deviations from the intended intervention beyond what would be expected in usual practice?	Probably No	No reported deviation
**4.2. If Y/PY to 4.1: Were these deviations from intended intervention unbalanced between groups and likely to have affected the outcome?**	Not applicable	
Risk of bias judgement	Low	Not reported deviation or significant drop in the participation
**Bias due to missing data**		
5.1 Were outcome data available for all, or nearly all, participants?	Yes	Attrition rate was low
5.2 Were participants excluded due to missing data on intervention status?	No	Not details on the dropped-out participants
5.3 Were participants excluded due to missing data on other variables needed for the analysis?	No	Not missing data

**5.4 If PN/N to 5.1, or Y/PY to 5.2 or 5.3: Are the proportion of participants and reasons for missing data similar across interventions?**	Not applicable	
**5.5 If PN/N to 5.1, or Y/PY to 5.2 or 5.3: Is there evidence that results were robust to the presence of missing data?**	Not applicable	
Risk of bias judgement	Low	
**Bias in measurement of outcomes**		
6.1 Could the outcome measure have been influenced by knowledge of the intervention received?	Probably yes	
6.2 Were outcome assessors aware of the intervention received by study participants?	No Information	
6.3 Were the methods of outcome assessment comparable across intervention groups?	Probably yes	
6.4 Were any systematic errors in measurement of the outcome related to intervention received?	Probably No	
Risk of bias judgement	Low	As it's a peer comparison study, knowledge of the outcome measures is apparent to all participants. No reported impact of the prior knowledge on the outcome
**Bias in selection of the reported result**		
Is the reported effect estimate likely to be selected, on the basis of the results, from...<br>7.1. ... multiple outcome measurements within the outcome domain?	Probably No	
7.2 ... multiple analyses of the intervention-outcome relationship?	Not information	Not access to the protocol to evaluate or pre-specify the methods
7.3 ... different subgroups?	No	
Risk of bias judgement	Moderate	
Overall bias	Moderate	
Tool: Jonathan AC Sterne, Julian PT Higgins, Roy G Elbers and Barney C Reeves. Risk Of Bias In Non-randomized Studies of Interventions (ROBINS-I): detailed guidance. October 2016. Available in: https://sites.google.com/site/riskofbiastool/welcome/home/current-version-of-robins-i		

**Title:** A multimodal intervention to decrease inappropriate outpatient antibiotic prescribing for upper respiratory tract infections in a large integrated healthcare system.		
**Study ID:** Davidson 2022 (13)	**Final decision**	**Comments**
**Domain 1: Bias due to confounding**		

1.1 Is there potential for confounding of the effect of intervention in this study?	Yes	Comments= seasonality in the data and implementation wash-in period
**If Y/PY to 1.1: determine whether there is a need to assess time-varying confounding:**<br>1.2. Was the analysis based on splitting participants' follow up time according to intervention received?<br>If N/PN, answer questions relating to baseline confounding (1.4 to 1.6)<br>If Y/PY, proceed to question 1.3.	Yes	Quote="*segmented regression models were constructed to compare level changes (ie, abrupt) and slope changes (ie, gradual) in antibiotic prescribing between preintervention and intervention periods*"
**If Y/PY to 1.1: determine whether there is a need to assess time-varying confounding:**<br>1.3. Were intervention discontinuations or switches likely to be related to factors that are prognostic for the outcome?<br>If N/PN, answer questions relating to baseline confounding (1.4 to 1.6)<br>If Y/PY, answer questions relating to both baseline and time-varying confounding (1.7 and 1.8)	Probably No	
1.4. Did the authors use an appropriate analysis method that<br>controlled for all the important confounding domains?	Yes	Quote= ordinary least-squares time-series regression models, sensitivity analyses to examine the influence of selecting different preintervention and intervention comparison months, with and without an implementation wash-in period, they chose the final model as the best reflection of the data and program rollout, while adjusting for seasonality in the data.
**1.5. If Y/PY to 1.4: Were confounding domains that were controlled for measured validly and reliably by the variables available in this study?**	Yes	
1.6. Did the authors control for any post-intervention variables that could have been affected by the intervention?	No	

1.7. Did the authors use an appropriate analysis method that adjusted for all the important confounding domains and for time-varying confounding?	Yes	
**1.8. If Y/PY to 1.7: Were confounding domains that were adjusted for measured validly and reliably by the variables available in this study?**	Yes	
Risk of bias judgement	Moderate	Overall, measurement of outcome was made at sufficient pre-intervention time points to permit characterization of pre-intervention trends and patterns. Confounding expected, all known important confounding domains appropriately measured and controlled
**Bias in selection of participants into the study**		
2.1. Was selection of participants into the study (or into the analysis) based on participant characteristics observed after the start of intervention? **If N/PN to 2.1: go to 2.4.**	Probably No	
**2.2. If Y/PY to 2.1: Were the post-intervention variables that influenced selection likely to be associated with intervention?**	Not applicable	
**2.3 If Y/PY to 2.2: Were the post-intervention variables that influenced selection likely to be influenced by the outcome or a cause of the outcome?**	Not applicable	
2.4. Do start of follow-up and start of intervention coincide for most participants?	Probably yes	
**2.5. If Y/PY to 2.2 and 2.3, or N/PN to 2.4: Were adjustment techniques used that are likely to correct for the presence of selection biases?**	Not applicable	
Risk of bias judgement	Low	For each ambulatory family medicine, start of follow up and start of intervention coincided.
**Bias in classification of interventions**		
3.1 Were intervention groups clearly defined?	Probably yes	the question relates to whether the population is clearly defined,
3.2 Was the information used to define intervention groups recorded at the start of the intervention?	Probably yes	For population-level intervention

3.3 Could classification of intervention status have been affected by knowledge of the outcome or risk of the outcome?	Probably no	
Risk of bias judgement	Low	
**Bias due to deviations from intended interventions**		
4.1. Were there deviations from the intended intervention beyond what would be expected in usual practice?	Probably no	
**4.2. If Y/PY to 4.1: Were these deviations from intended intervention unbalanced between groups and likely to have affected the outcome?**	Not applicable	
Risk of bias judgement	Low	Any deviations from intended intervention reflected usual practice
**Bias due to missing data**		
5.1 Were outcome data available for all, or nearly all, participants?	Yes	
5.2 Were participants excluded due to missing data on intervention status?	No	
5.3 Were participants excluded due to missing data on other variables needed for the analysis?	No	
**5.4 If PN/N to 5.1, or Y/PY to 5.2 or 5.3: Are the proportion of participants and reasons for missing data similar across interventions?**	Not applicable	
**5.5 If PN/N to 5.1, or Y/PY to 5.2 or 5.3: Is there evidence that results were robust to the presence of missing data?**	Not applicable	
Risk of bias judgement	Low	
**Bias in measurement of outcomes**		
6.1 Could the outcome measure have been influenced by knowledge of the intervention received?	Probably no	
6.2 Were outcome assessors aware of the intervention received by study participants?	Probably yes	
6.3 Were the methods of outcome assessment comparable across intervention groups?	Yes	Comment= methods of outcome assessment were comparable before and after the intervention
6.4 Were any systematic errors in measurement of the outcome related to intervention received?	Probably no	
Risk of bias judgement	Low	
**Bias in selection of the reported result**		

Is the reported effect estimate likely to be selected, on the basis of the results, from...<br>7.1. ... multiple outcome measurements within the outcome domain?	Probably no	
7.2 ... multiple analyses of the intervention-outcome relationship?	Not information	Not access to the protocol to evaluate or pre-specify the methods
7.3 ... different subgroups?	Probably yes	Comment= it did not report the result of regression analysis by subgroup of antibiotic class.
Risk of bias judgement	Moderate	
Overall bias	Moderate	
Tool: Jonathan AC Sterne, Julian PT Higgins, Roy G Elbers and Barney C Reeves. Risk Of Bias In Non-randomized Studies of Interventions (ROBINS-I): detailed guidance. October 2016. Available in: https://sites.google.com/site/riskofbiastool/welcome/home/current-version-of-robins-i<br>Sterne J, Hernán M, McAleenan A, Reeves B, Higgins J. 25.5 Risk of bias in uncontrolled before-after studies (including interrupted time series). In: Cochrane Handbook for Systematic Reviews of Interventions version 63 (updated February 2022) [Internet]. 2022. p. section-25-5. Available from: https://training.cochrane.org/handbook/current/chapter-25#section-25-5		

References

1. Prior M, Elouafkaoui P, Elders A, Young L, Duncan EM, Newlands R, et al. Evaluating an audit and feedback intervention for reducing antibiotic prescribing behaviour in general dental practice (the RAPiD trial): A partial factorial cluster randomised trial protocol. Implement Sci. 2014;9(1):1–10.

2. Elouafkaoui P, Young L, Newlands R, Duncan EM, Elders A, Clarkson JE, et al. An Audit and Feedback Intervention for Reducing Antibiotic Prescribing in General Dental Practice: The RAPiD Cluster Randomised Controlled Trial. PLoS Med. 2016;13(8):1–19.

3. Curtis HJ, Bacon S, Croker R, Walker AJ, Perera R, Hallsworth M, et al. Evaluating the impact of a very low-cost intervention to increase practices' engagement with data and change prescribing behaviour: a randomized trial in English primary care. Fam Pract. 2021;38(4):373–80.

4. Linder JA, Schnipper JL, Tsurikova R, Yu DT, Volk LA, Melnikas AJ, et al. Electronic health record feedback to improve antibiotic prescribing for acute respiratory infections. Am J Manag Care. 2010;16(12 Suppl HIT):311–9.

5. Shen XR, Lu M, Feng R, Cheng J, Chai J, Xie M, et al. Web-based just-in-time information and feedback on antibiotic use for village doctors in rural Anhui, China: Randomized controlled trial. J Med Internet Res. 2018;20(2).

6. Du Yan L, Dean K, Park D, Thompson J, Tong I, Liu C, et al. Education vs Clinician Feedback on Antibiotic Prescriptions for Acute Respiratory Infections in Telemedicine: a Randomized Controlled Trial. J Gen Intern Med. 2021;36(2):305–12.

7. Daneman N, Lee SM, Bai H, Bell CM, Bronskill SE, Campitelli MA, et al. Population-Wide Peer Comparison Audit and Feedback to Reduce Antibiotic Initiation and Duration in Long-Term Care Facilities with Embedded Randomized Controlled Trial. Clin Infect Dis. 2021;73(6):e1296–304.

8. Laur C, Sribaskaran T, Simeoni M, Desveaux L, Daneman N, Mulhall C, et al. Improving antibiotic initiation and duration prescribing among nursing home physicians using an audit and feedback intervention: A theory-informed qualitative analysis. BMJ Open Qual. 2021;10(1):1–9.

9. Hemkens LG, Saccilotto R, Reyes SL, Glinz D, Zumbrunn T, Grolimund O, et al. Personalized prescription feedback to reduce antibiotic overuse in primary care : rationale and design of a nationwide pragmatic randomized trial. BMC Infect Dis [Internet]. 2016;1–8. Available from: http://dx.doi.org/10.1186/s12879-016-1739-0

10. Hemkens LG, Saccilotto R, Reyes SL, Glinz D, Zumbrunn T, Grolimund O, et al. Personalized Prescription Feedback Using Routinely Collected Data to Reduce Antibiotic Use in Primary Care: A Randomized Clinical Trial. JAMA Intern Med [Internet]. 2017;177(2):176–83. Available from: https://doi.org/10.1001/jamainternmed.2016.8040

11. Chang Y, Sangthong R, McNeil EB, Tang L, Chongsuvivatwong V. Effect of a computer network-based feedback program on antibiotic prescription rates of primary care physicians: A cluster randomized crossover-controlled trial. J Infect Public Health [Internet]. 2020;13(9):1297–303. Available from: https://doi.org/10.1016/j.jiph.2020.05.027

12. Jones GF, Fabre V, Hinson J, Levin S, Toerper M, Townsend J, et al. Improving antimicrobial prescribing for upper respiratory infections in the emergency department: Implementation of peer comparison with behavioral feedback. Antimicrob Steward Healthc Epidemiol. 2021;1(1):1–6.

13. Davidson LE, Gentry EM, Priem JS, Kowalkowski M, Spencer MD. A multimodal intervention to decrease inappropriate outpatient antibiotic prescribing for upper respiratory tract infections in a large integrated healthcare system. Infect Control Hosp Epidemiol. 2022;1–8.
