# Supplementary material for: The Effectiveness of Interactive Dashboards to Optimise Antibiotic Prescribing in Primary Care: A Systematic Review
Source: Antibiotics (Basel). 2023 Jan 10;12(1):136. doi: 10.3390/antibiotics12010136 (PMC9854857; doi:10.3390/antibiotics12010136)
Supplement: Supplementary file 1 [file antibiotics-12-00136-s001.zip › Table S5 - GRADE assessment.pdf]

Table S5. GRADE (Grading of Recommendation, Assessment, Development and Evaluation) approach to assess the quality of evidence for the primary outcome (overall change of antibiotic prescriptions).

**GRADE domain**	**Judgement**	**Concerns about certainty domains**
Methodological limitations of the studies	One out of eight studies [1] had a low risk of bias (RoB) overall. Also, two studies had a high RoB due to the randomisation process, measurement of the outcome, selection of the reported result, and bias arising from period and carryover effects [2,3]. The controlled before and after study presented moderate RoB judgement due to confounding [4]. It was judged to have serious methodological limitations.	Serious
Indirectness	There are no differences in population and settings. The interventions and comparators differed among studies (delivered method and combinations of interventions). The evidence was judged to have borderline indirectness due to some variability in the intervention and comparisons.	Not serious, borderline
Imprecision	The results of this primary outcome (overall change of antibiotic prescriptions) in all studies do not have a wide confidence interval around the effect estimate (see figure 2 and 3, and appendix 5). It was judged the evidence to have no serious imprecision.	Not serious
Inconsistency	The direction and magnitude of impact varied across the different studies. Overall, the results of five studies showed a reduction in AP [2,4–7]. Three studies found no significant between-group differences [1,3,8], however, these studies used no intervention or usual care as a comparison. Some subgroups of these three studies demonstrated a reduction of AP [1,8]. It was judged the evidence to have serious inconsistency.	Serious
Publication bias	It did not strongly suspect publication bias because Search methods for the identification of studies were comprehensive and exhaustive. Also, it included studies with both negative and positive effects.	Not suspected
Overall Certainty of Evidence	Impact: Interactive dashboards may reduce AP in primary care; however, it is relevant to consider combination interventions, have key strategies in the implementation process and include dashboard engagement strategies.	⊕⊕OO Low

Source: Rating the certainty in evidence in the absence of a single estimate of effect [9].

References

1. Hemkens, L.G.; Saccilotto, R.; Reyes, S.L.; Glinz, D.; Zumbrunn, T.; Grolimund, O.; Gloy, V.; Raatz, H.; Widmer, A.; Zeller, A.; et al. Personalized Prescription Feedback Using Routinely Collected Data to Reduce Antibiotic Use in Primary Care: A Randomized Clinical Trial. *JAMA Intern. Med.* **2017**, *177*, 176–183, doi:10.1001/jamainternmed.2016.8040.

2. Shen, X.R.; Lu, M.; Feng, R.; Cheng, J.; Chai, J.; Xie, M.; Dong, X.; Jiang, T.; Wang, D. Web-Based Just-in-Time Information and Feedback on Antibiotic Use for Village Doctors in Rural Anhui, China: Randomized Controlled Trial. *J. Med. Internet Res.* **2018**, *20*, doi:10.2196/jmir.8922.

3. Chang, Y.; Sangthong, R.; McNeil, E.B.; Tang, L.; Chongsuvivatwong, V. Effect of a Computer Network-Based Feedback Program on Antibiotic Prescription Rates of Primary Care Physicians: A Cluster Randomized Crossover-Controlled Trial. *J. Infect. Public Health* **2020**, *13*, 1297–1303, doi:10.1016/j.jiph.2020.05.027.

4. Jones, G.F.; Fabre, V.; Hinson, J.; Levin, S.; Toerper, M.; Townsend, J.; Cosgrove, S.E.; Saheed, M.; Klein, E.Y. Improving Antimicrobial Prescribing for Upper Respiratory Infections in the Emergency Department: Implementation of Peer Comparison with Behavioral Feedback. *Antimicrob. Steward. Healthc. Epidemiol.* **2021**, *1*, 1–6, doi:10.1017/ash.2021.240.

5. Du Yan, L.; Dean, K.; Park, D.; Thompson, J.; Tong, I.; Liu, C.; Hamdy, R.F. Education vs Clinician Feedback on Antibiotic Prescriptions for Acute Respiratory Infections in Telemedicine: A Randomized Controlled Trial. *J. Gen. Intern. Med.* **2021**, *36*, 305–312, doi:10.1007/s11606-020-06134-0.

6. Curtis, H.J.; Bacon, S.; Croker, R.; Walker, A.J.; Perera, R.; Hallsworth, M.; Harper, H.; Mahtani, K.R.; Heneghan, C.; Goldacre, B. Evaluating the Impact of a Very Low-Cost Intervention to Increase Practices' Engagement with Data and Change Prescribing Behaviour: A Randomized Trial in English Primary Care. *Fam. Pract.* **2021**, *38*, 373–380, doi:10.1093/fampra/cmaa128.

7. Elouafkaoui, P.; Young, L.; Newlands, R.; Duncan, E.M.; Elders, A.; Clarkson, J.E.; Ramsay, C.R. An Audit and Feedback Intervention for Reducing Antibiotic Prescribing in General Dental Practice: The RAPiD Cluster Randomised Controlled Trial. *PLoS Med.* **2016**, *13*, 1–19, doi:10.1371/journal.pmed.1002115.

8. Linder, J.A.; Schnipper, J.L.; Tsurikova, R.; Yu, D.T.; Volk, L.A.; Melnikas, A.J.; Palchuk, M.B.; Olsha-Yehiav, M.; Middleton, B. Electronic Health Record Feedback to Improve Antibiotic Prescribing for Acute Respiratory Infections. *Am. J. Manag. Care* **2010**, *16*, 311–319.

9. Murad, M.H.; Mustafa, R.A.; Schünemann, H.J.; Sultan, S.; Santesso, N. Rating the Certainty in Evidence in the Absence of a Single Estimate of Effect. *Evid. Based. Med.* **2017**, *22*, 85–87, doi:10.1136/ebmed-2017-110668.
