# Supplementary material for: The Effectiveness of Interactive Dashboards to Optimise Antibiotic Prescribing in Primary Care: A Systematic Review
Source: Antibiotics (Basel). 2023 Jan 10;12(1):136. doi: 10.3390/antibiotics12010136 (PMC9854857; doi:10.3390/antibiotics12010136)
Supplement: Supplementary file 1 [file antibiotics-12-00136-s001.zip › Table S6 - Electronic search report.pdf]

Table S6. Evidence search report in electronic databases

Electronic search report No. 1	
**Electronic database**	Ovid MEDLINE(R) and Epub Ahead of Print, In-Process, In-Data-Review & Other Non-Indexed Citations, Daily and Versions 1946 to August 12, 2022
**Platform**	OVID
**Date of search**	15-08-2022
**Range of date**	1946 to August 12, 2022
**Restriction of language**	None
**Other limits**	None
**Search strategy (results)**	1. exp general Practitioners/ (9922)<br>2. general practice physician*.tw. (122)<br>3. general practitioner*.tw. (55067)<br>4. GPs.tw. (29461)<br>5. exp Family Practice/ or general practice.tw. (87967)<br>6. (family and (practice* or doctor*)).tw. (68669)<br>7. (Clinician* or physician*).tw. (690042)<br>8. primary care practice*.tw. (7073)<br>9. exp Primary Health Care/ (184933)<br>10. 1 or 2 or 3 or 4 or 5 or 6 or 7 or 8 or 9 (991469)<br>11. exp electronic Health Records/ (26327)<br>12. ((electronic or computeriz#ed) and (health or medical) and record*).tw. (54070)<br>13. exp User-Computer Interface/ or computer interface.tw. (42547)<br>14. ((User-computer or User computer) and Interface*).tw. (15)<br>15. Dashboard*.tw. (2186)<br>16. visual analytic*.tw. (804)<br>17. exp Health Information System/ or medical information system.tw. (2061)<br>18. Health Information System* Data.tw. (68)<br>19. Virtual Desktop Infrastructure.tw. (5)<br>20. data visualisation*.tw. (182)<br>21. exp Computer Graphics/ (41360)<br>22. computer graphic*.tw. (2057)<br>23. exp clinical audit/ and exp medical audit/ (17631)<br>24. visual health record.tw. (0)<br>25. Interactive visualization tool*.tw. (137)<br>26. 11 or 12 or 13 or 14 or 15 or 16 or 17 or 18 or 19 or 20 or 21 or 22 or 23 or 24 or 25 (165125)<br>27. 10 and 26 (25591)<br>28. exp drug Prescriptions/ (37649)<br>29. ((Drug or antibiotic) and Prescri*).tw. (76072)<br>30. exp inappropriate Prescribing/ or prescribing error.tw. (4554)<br>31. ((Inappropriate or over) and Prescri*).tw. (47890)

	32. 28 or 29 or 30 or 31 (133694)<br>33. 27 and 32 (1739)
**Number of references found**	1739
**Electronic search report No. 2**	
**Electronic database**	Embase
**Platform**	Elsevier
**Date of search**	15-08-2022
**Range of date**	None
**Restriction of language**	None
**Other limits**	None
**Search strategy (results)**	1 'general practitioner'/exp OR 'general practitioner*':ab,ti (144361)<br>2 'general practice physician*':ab,ti (154)<br>3 'gps':ab,ti (40270)<br>4 'general practice'/exp (89390)<br>5 'family practice':ab,ti (8499)<br>6 family:ab,ti AND (practice*:ab,ti OR doctor*:ab,ti) (93761)<br>7 clinician*:ab,ti OR physician*:ab,ti (989243)<br>8 'primary care practice*':ab,ti (9140)<br>9 'primary health care'/exp (194701)<br>10 #1 OR #2 OR #3 OR #4 OR #5 OR #6 OR #7 OR #8 OR #9 (1349236)<br>11 'electronic health record'/exp (31630)<br>12 (electronic:ab,ti OR computeriz?ed:ab,ti) AND (health:ab,ti OR medical:ab,ti) AND record*:ab,ti (98906)<br>13 'computer interface'/exp OR 'user-computer interface':ab,ti (34620)<br>14 ('user computer':ab,ti OR user:ab,ti) AND computer:ab,ti AND interface*:ab,ti (3755)<br>15 dashboard*:ab,ti (3832)<br>16 'visual analytics':ab,ti (640)<br>17 'medical information system'/exp OR 'health information system':ab,ti (24309)<br>18 'health information system* data':ab,ti (80)<br>19 'virtual desktop infrastructure':ab,ti (6)<br>20 'data visualization'/exp (1839)<br>21 'computer graphics'/exp (10788)<br>22 'computer graphic*':ab,ti (2181)<br>23 'clinical audit'/exp (53041)<br>24 'medical audit':ab,ti (986)<br>25 'visual health record' (0)<br>26 'interactive visualization tool*':ab,ti (128)<br>27 #11 OR #12 OR #13 OR #14 OR #15 OR #16 OR #17 OR #18 OR #19 OR #20 OR #21 OR #22 OR #23 OR #24 OR #25 OR #26 (230562)<br>28 #10 AND #27 (45781)

	29 'drug prescriptions':ab,ti (2560)<br>30 (drug:ab,ti OR antibiotic:ab,ti) AND prescri*:ab,ti (128368)<br>31 'prescribing error'/exp OR 'inappropriate prescribing':ab,ti (8478)<br>32 (inappropriate:ab,ti OR over:ab,ti) AND prescri*:ab,ti (89868)<br>33 #29 OR #30 OR #31 OR #32 (189106)<br>34 #28 AND #33 (3758)<br>35 #34 AND [embase]/lim NOT ([embase]/lim AND [medline]/lim) (2217)
**Number of references found**	2217
**Electronic search report No. 3**	
**Electronic database**	EBM Reviews - Cochrane Central Register of Controlled Trials
**Platform**	OVID
**Date of search**	15-08-2022
**Range of date**	None
**Restriction of language**	None
**Other limits**	None
**Search strategy (results)**	1 general Practitioner*.mp. (8208)<br>2 general practice physician*.mp. (7)<br>3 GPs.mp. (3301)<br>4 (Family Practice or general practice).mp. (7213)<br>5 (family and (practice* or doctor*)).mp. (9786)<br>6 (Clinician* or physician*).mp. (77166)<br>7 primary care practice*.mp. (1802)<br>8 Primary Health Care.mp. (7725)<br>9 1 or 2 or 3 or 4 or 5 or 6 or 7 or 8 (97865)<br>10 electronic Health Records.mp. (1251)<br>11 ((electronic or computeriz#ed) and (health or medical) and record*).mp. (7314)<br>12 (User-Computer Interface or computer interface).mp. (1824)<br>13 ((User-computer or User computer) and Interface*).mp. (1377)<br>14 Dashboard*.mp. (343)<br>15 visual analytic*.mp. (8)<br>16 (Health Information Systems or medical information system).mp. (328)<br>17 Health Information System* Data.mp. (5)<br>18 Virtual Desktop Infrastructure.mp. (0)<br>19 data visualisation*.mp. (4)<br>20 computer graphic*.mp. (215)<br>21 clinical audit.mp. (559)<br>22 medical audit.mp. (413)

	23 visual health record.mp. (0)<br>24 Interactive visualization tool*.mp. (0)<br>25 10 or 11 or 12 or 13 or 14 or 15 or 16 or 17 or 18 or 19 or 20 or 21 or 22 or 23 or 24 (10728)<br>26 9 and 25 (3547)<br>27 drug Prescriptions.mp. (651)<br>28 ((Drug or antibiotic) and Prescri*).mp. (22240)<br>29 (inappropriate Prescribing or prescribing error).mp. (464)<br>30 ((Inappropriate or over) and Prescri*).mp. (12307)<br>31 27 or 28 or 29 or 30 (27717)<br>32 26 and 31 (574)
**Number of references found**	574
**Electronic search report No. 4**	
**Electronic database**	SCOPUS
**Platform**	SCOPUS
**Date of search**	15-08-2022
**Range of date**	None
**Restriction of language**	None
**Other limits**	None
**Search strategy (results)**	((TITLE-ABS("general practitioner*" OR "general practice physician*" OR "GPs" OR "general practice" OR "family practice" OR ("family" AND ("practice*" OR "doctor*")) OR "clinician*" OR "physician*" OR "primary care practice*" OR "primary health care")) AND (TITLE-ABS("electronic health record" OR (("electronic" OR "computeriz?ed") AND ("health" OR "medical") AND "record*") OR "User-Computer Interface" OR "computer interface" OR (("User-computer" OR "User computer") AND "Interface*") OR "Dashboard*" OR "visual analytic*" OR "Health Information System*" OR "medical information system*" OR "Health Information System* Data" OR "Virtual Desktop Infrastructure" OR "data visualisation*" OR "computer graphic*" OR "clinical audit" OR "medical audit" OR "visual health record" OR "Interactive visualization tool*"))) AND (TITLE-ABS("drug Prescriptions" OR (("Drug" OR "antibiotic") AND "Prescri*") OR "inappropriate Prescribing" OR "prescribing error" OR (("Inappropriate" OR "over") AND "Prescri*"))) AND (LIMIT-TO (DOCTYPE,"ar"))
**Number of references found**	1087
**Electronic search report No. 5**	
**Electronic database**	Web of Science Core Collection

**Platform**	Wed of Science
**Date of search**	15-08-2022
**Range of date**	None
**Restriction of language**	None
**Other limits**	None
**Search strategy (results)**	1. AB=(general practitioner* OR general practice physician* OR GPs OR general practice OR family practice OR (family AND (practice* OR doctor*)) OR clinician* OR physician* OR primary care practice* OR primary health care) (1,339,524)<br>2. AB=(electronic health record OR ((electronic OR computeriz*) AND (health OR medical) AND record*) OR User-Computer Interface OR computer interface OR ((User-computer OR User computer) AND Interface*) OR Dashboard* OR visual analytic* OR Health Information System* OR medical information system* OR Health Information System* Data OR Virtual Desktop Infrastructure OR data visualisation* OR computer graphic* OR clinical audit OR medical audit OR visual health record OR Interactive visualization tool*) (792,343)<br>3. #2 AND #1 (64,353)<br>4. AB=(drug Prescriptions OR ((Drug OR antibiotic) AND Prescri*) OR inappropriate Prescribing OR prescribing error OR ((Inappropriate OR over) AND Prescri*)) (175,701)<br>5. #4 AND #3 and Articles (Document Types) and Web of Science Core Collection (Database) (2,501)
**Number of references found**	2501
**Electronic search report No. 6**	
**Electronic database**	LILACS
**Platform**	Virtual Health Library https://bvsalud.org/en/
**Date of search**	15-08-2022
**Range of date**	None
**Restriction of language**	None
**Other limits**	None
**Search strategy (results)**	(general practitioner*) OR (general practice physician*) OR (GPs) OR (general practice) OR (family practice) OR ((family) AND ((practice*) OR (doctor*))) OR (clinician*) OR (physician*) OR (primary care practice*) OR (primary health care)<br><br>AND<br><br>(electronic health record) OR (((electronic) OR (computerize*)) AND ((health) OR (medical)) AND (record*)) OR (User Computer

	Interface) OR (computer interface) OR (Dashboard*) OR (visual analytic*) OR (Health Information System*) OR (medical information system*) OR (Health Information System* Data) OR (Virtual Desktop Infrastructure) OR (data visualisation*) OR (computer graphic*) OR (clinical audit) OR (medical audit) OR (visual health record) OR (Interactive visualization tool*)<br><br>AND<br><br>(drug Prescriptions) OR (((Drug) OR (antibiotic)) AND (Prescri*)) OR (inappropriate Prescribing) OR (prescribing error) OR (((Inappropriate) OR (over)) AND (Prescri*))
**Number of references found**	106
